# An improved and efficient method of *Agrobacterium* syringe infiltration for transient transformation and its application in the elucidation of gene function in poplar

**DOI:** 10.1186/s12870-021-02833-w

**Published:** 2021-01-21

**Authors:** Lin Zheng, Jixiu Yang, Yajuan Chen, Liping Ding, Jianhua Wei, Hongzhi Wang

**Affiliations:** 1 Beijing Agro-Biotechnology Research Center, Beijing Academy of Agricultural and Forestry Sciences, No. 9, Shuguang Huayuan Middle Road, Haidian District, Beijing, 100097 People’s Republic of China; 2 College of Bioscience and Resources Environment, Beijing University of Agriculture, No. 7, Beinong Road, Huilongguan, Changping District, Beijing, 102206 People’s Republic of China

**Keywords:** Transient expression, Syringe *Agrobacterium* infiltration, Poplar, Transgenic poplar, Secondary wall formation

## Abstract

**Background:**

Forest trees have important economic and ecological value. As a model tree, poplar has played a significant role in elucidating the molecular mechanisms underlying tree biology. However, a lack of mutant libraries and time-consuming stable genetic transformation processes severely limit progress into the functional characterization of poplar genes. A convenient and fast transient transformation method is therefore needed to enhance progress on functional genomics in poplar.

**Methods:**

A total of 11 poplar clones were screened for amenability to syringe infiltration. Syringe infiltration was performed on the lower side of the leaves of young soil-grown plants. Transient expression was evaluated by visualizing the reporters β-glucuronidase (GUS) and green fluorescent protein (GFP). The experimental parameters of the syringe agroinfiltration were optimized based on the expression levels of the reporter luciferase (LUC). Stably transformed plants were regenerated from transiently transformed leaf explants through callus-induced organogenesis. The functions of *Populus* genes in secondary cell wall-thickening were characterized by visualizing lignin deposition therein after staining with basic fuchsin.

**Results:**

We greatly improved the transient transformation efficiency of syringe *Agrobacterium* infiltration in poplar through screening for a suitable poplar clone from a variety of clones and optimizing the syringe infiltration procedure. The selected poplar clone, *Populus davidiana* × *P. bolleana*, is amenable to *Agrobacterium* syringe infiltration, as indicated by the easy diffusion of the bacterial suspension inside the leaf tissues. Using this technique, we localized a variety of poplar proteins in specific intracellular organelles and illustrated the protein–protein and protein–DNA interactions. The transiently transformed leaves could be used to generate stably transformed plants with high efficiency through callus induction and differentiation processes. Furthermore, transdifferentiation of the protoxylem-like vessel element and ectopic secondary wall thickening were induced in the agroinfiltrated leaves via the transient overexpression of genes associated with secondary wall formation.

**Conclusions:**

The application of *P. davidiana* × *P. bolleana* in *Agrobacterium* syringe infiltration provides a foundation for the rapid and high-throughput functional characterization of *Populus* genes in intact poplar plants, including those involved in wood formation, and provides an effective alternative to *Populus* stable genetic transformation.

**Supplementary Information:**

The online version contains supplementary material available at 10.1186/s12870-021-02833-w.

## Background

Forest trees have important economic and ecological value and have thus been the focus of studies on fundamental issues in tree biology. Due to several biological advantages, including a rapid growth rate, small genome size, and ease of clonal propagation and genetic transformation [1, 2], poplars have been used as a model to evaluate the cellular and molecular mechanisms underlying the distinct biology of trees, such as extensive secondary growth and a perennial habit. As the genomes of several *Populus* species have been sequenced [3, 4], the elucidation of poplar gene function can provide a foundation for the genetic modification of forest trees. However, the lack of mutant libraries and the time-consuming stable genetic transformation process severely limit progress on functional genomics in poplar. Therefore, a convenient and rapid transient transformation method in poplar is required and will enhance high-throughput functional analyses of *Populus* genes.

Transient gene expression has become a powerful tool for studying gene function due to its simplicity, speed, and efficiency over stable genetic transformation [5, 6]. Currently, three transient transformation techniques—biolistic bombardment, protoplast transformation, and *Agrobacterium* infiltration—have been widely used in gene function analysis, such as in the subcellular localization of proteins of interest, interaction between proteins, transaction of transcription factors, and gene overexpression or repression [7, 8]. However, several disadvantages in the methodologies of biolistic bombardment and protoplast transformation limit their application in high-throughput analyses of gene function. For example, biolistic bombardment is relatively expensive due to the requirement of gold microparticles and a costly gene gun system [9] and can cause genome damage in rice and maize [10]. Protoplast transformation is not suitable for the analysis of macromolecule trafficking between cells. Moreover, the removal of cell walls during protoplast preparation causes an alteration in the subcellular organization of the cytoskeleton and endoplasmic reticulum (ER), which will compromise the experimental results for the proteins localized in these compartments [9]. These limitations in biolistic bombardment and protoplast transformation make *Agrobacterium* infiltration a preferred method for transient transformation. As a result, *Agrobacterium* infiltration has become the favorable gene delivery method for transient expression in plants [11, 12].

*Agrobacterium* infiltration, by which the suspension culture of agrobacterial cells is infiltrated into the organs of an intact plant, provides a rapid and efficient way to transiently express foreign genes *in planta* [11, 13]. Due to the high efficiency of T-DNA transfer, the power of this technique has been described in many plant species, such as *Nicotiana benthamiana* [14], *Arabidopsis thaliana* [15, 16], *Medicago sativum* [17], and *Solanum lycopersicum* [16, 18]. It has been widely used for transgenic complementation [19], transaction assays in intact plants [20], plant-pathogen interaction [21, 22], promoter analysis *in planta* [5], identification of the biological function of genes [23], protein production [24], a variety of transient expression assays to study protein localization [25, 26], and protein–protein interaction [27]. Throughout the years, several agroinfiltration methods have been developed, including syringe infiltration (agroinjection) [13, 16, 18], vacuum infiltration [28, 29], and agrodrench (soil adjacent to the plant roots is drenched with *Agrobacterium* suspension) [30]. Among these methods, vacuum infiltration has the disadvantage of being complicated to operate and is also associated with variable results and typically weak expression, which are probably due to uneven tissue permeation by the *Agrobacterium* suspension [16]. Agrodrench normally works with the genes expressed in the roots [30]. Syringe infiltration is the simplest and most efficient method of agroinfiltration for gene function analyses. It allows multiple transient expression assays to be performed on a single leaf [12], which facilitates the optimization of experimental parameters that potentially influence the protein expression efficiency. Although only a few plant species are naturally amenable to syringe infiltration [11], with great effort, syringe infiltration has been successfully applied to *Arabidopsis* [16], tobacco [31], onion [32], potato [33], citrus [34], tomato [16], grape [35], and lettuce [16]. However, this simple and highly efficient technique has not been applied in poplar, probably due to the inability of the *Agrobacterium* suspension to diffuse within the leaves. In fact, the high level of transient expression in agroinfiltration is highly dependent on the ability of the bacterial suspension to distribute widely inside the leaf tissue once it crosses the epidermal barrier [17]. The ease of spread of the *Agrobacterium* suspension inside the leaf makes *N. benthamiana* the most popular host plant for *Agrobacterium* syringe infiltration. Conversely, the limited spread of the *Agrobacterium* suspension from leaf vein networks makes this method fail in hybrid aspen *Populus tremula* × *P. tremuloides* [29]. It is worth mentioning that the ability of the *Agrobacterium* suspension to diffuse inside the leaf differs among cultivars within species, as shown in grapevine [35] and potato plants [33]. Therefore, via the wide screening of poplar clones, it could be possible to enhance the syringe infiltration method in certain clones.

In this study, we evaluated the responses of 11 different poplar clones at different development stages to *Agrobacterium* syringe infiltration and found that aspen hybrid *Populus davidiana* × *bolleana* was the most amenable to *Agrobacterium* syringe infiltration, with the bacterial suspension spreading easily inside the leaf tissue after infiltration. We optimized several experimental parameters affecting syringe agroinfiltration and achieved high levels of transient gene expression *in planta*. We applied this transient transformation method to characterize *Populus* genes in intact poplar plants, including those involved in the biosynthesis of the secondary cell wall (SCW), and demonstrated its potential to dissect the molecular mechanisms regulating SCW biosynthesis in poplar. Additionally, we also developed a method to generate stably transformed poplar lines by using the agroinfiltrated leaves as explants, which can be conveniently used for functional characterization of those genes needed to be further studied in cell types other than the leaf epidermis. Moreover, in this study, we disclosed for the first time that the amenability of a plant to syringe agroinfiltration is associated with the volume of intercellular air spaces and the arrangement of the mesophyll cells inside the leaves.

## Results

### Screening of poplar clones for *Agrobacterium* syringe infiltration

**Fig. 1:**
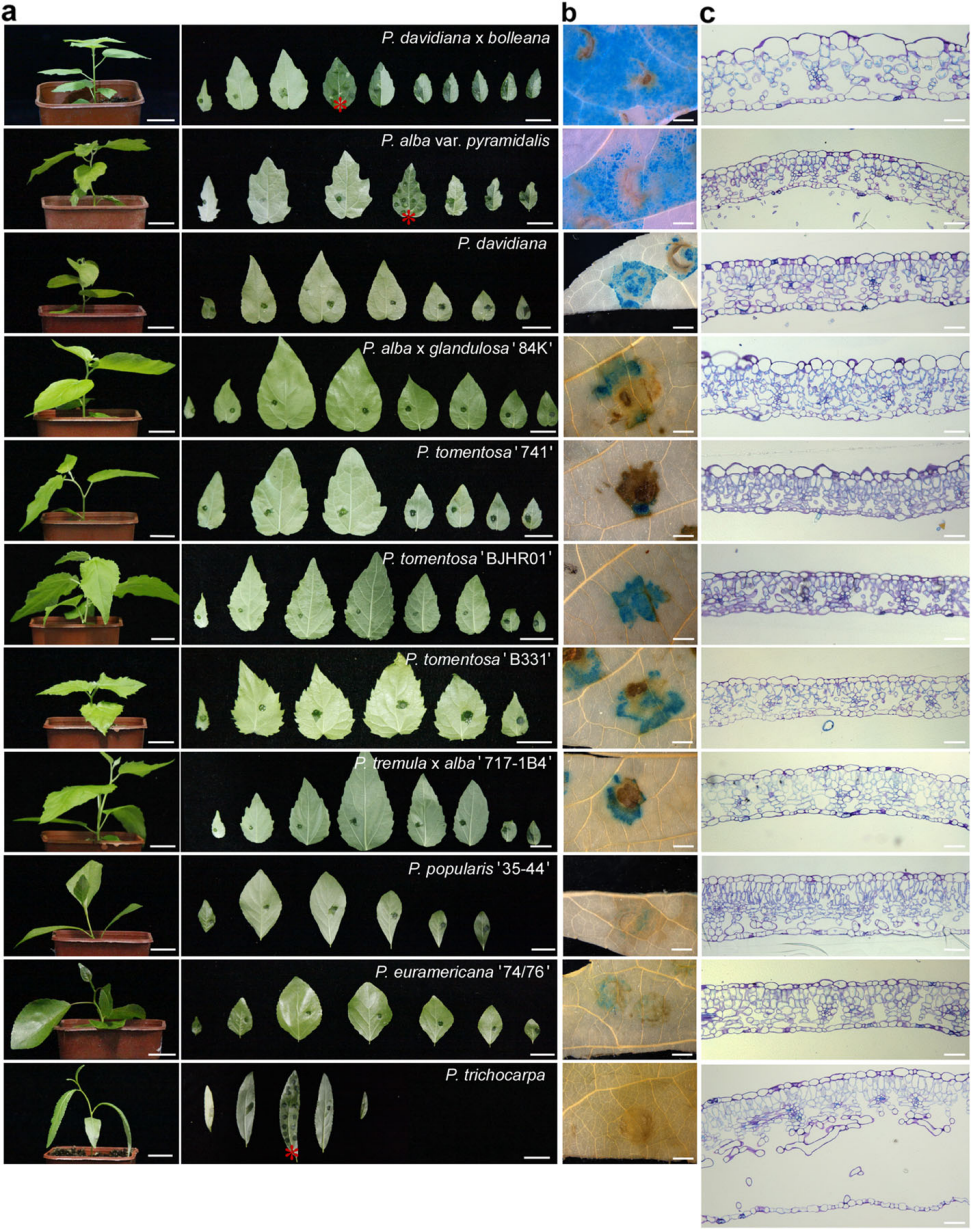
The spreadability of the agrobacterial suspension and expression of reporters in the tested poplar clones. The transformations were carried out using *A. tumefaciens* EHA105, which was suspended in the infiltrated medium [10 mM MgCl_2_, 5 mM MES-KOH (pH 5.6) and 0.2 mM Acetosyringone (AS)]. **a** The spreadability of the agrobacterial suspension in the leaves of the tested poplar clones. Agroinfiltration was performed on leaves from leaf Plastochron index (LPI) 1 down to the last one (shown sequentially from left to right). The bacterial suspension spread well in clones *P. davidiana* × *bolleana*, *P. alba var. pyramidalis*, and *P. trichocarpa*, with the best performance observed in the leaves LPI 4 of *P. davidiana* × *bolleana*, LPI 4 of *P. alba var. pyramidalis*, and LPI 3 of *P. trichocarpa*, as indicated by the red stars. In contrast, the bacterial suspension was shown to be limited to a very small region in all the manipulated leaves in the other clones. The suspension was clearly delineated by leaf veins in leaves LPI 3 of clones *P. tomentosa* ‘B331’ and *P. popularis* ‘35–44’. Bars = 2 cm. **b** The GUS staining in the leaves of the tested clones. All infiltrated leaves were stained, and the representative images are shown. Bars = 2 mm. **c** The interior structure of the full-expanded leaves of the tested clones. Five-micrometer-thick sections were stained with TBO and observed using a Leica DM 5500 B light microscope. The clones *P. davidiana* × *bolleana*, *P. alba var. pyramidalis*, and *P. trichocarpa* showed larger intercellular air spaces inside the leaves compared to the other clones, in which the air spaces were smaller and more compartmented. The mesophyll cells were arranged randomly and loosely within the leaves in the clone *P. davidiana* × *bolleana*

To enhance the syringe infiltration method for transient assay in poplar, a total of 11 poplar clones, including four white poplar clones (*P. alba var. pyramidalis*, i.e., *P. bolleana*, *P. tomentosa* ‘BJHR01’, *P. tomentosa* ‘741’, and *P. tomentosa* ‘B331’), three aspen or hybrid aspen (*P. davidiana*, *P. alba* × *glandulosa* ‘84 K’, and *P. tremula* × *alba* ‘INRA 717-1B4’), one aspen hybrid (*P. davidiana* × *bolleana*), two cottonwood (*P. euramericana* ‘74/76’ and *P. trichocarpa*), and one *P. popularis* ‘35–44’, were screened for amenability to syringe infiltration. We chose *Agrobacterium* strain EHA105, which is widely used in stable genetic transformation in *Populus* species [36, 37], for the initial evaluation. This strain contains the reporter binary vector *Super*:*GFP*-*Flag* or CaMV *35S*:*GUS*-*intro* for easy visualization of transient expression. The Super promoter consists of the transcriptional activating elements of octopine synthase and mannopine synthase2’, as well as the minimal promoter [38]. By using a syringe without a needle, *Agrobacterium* suspensions with an optical density (OD) of 1 in the infiltration medium [10 mM MgCl_2_, 5 mM MES-KOH (pH 5.6) and 0.2 mM Acetosyringone (AS)] were forced into the abaxial epidermis of fully expanded leaves of soil-grown plants after one month of growth. The response of the plants to syringe agroinfiltration was quite different among all the tested clones. The infiltrated bacterial suspension spread well inside the leaves of clones *P. davidiana* × *bolleana*, *P. alba var. pyramidalis*, and *P. trichocarpa* (Fig. 1a). However, in the other clones, the suspension was limited to a very small region, sometimes only as large as the size of the syringe tip. We noticed that the vein networks played roles in limiting the spread of the agrobacterial suspension in those clones, which was demonstrated by the delineated suspension in leaves LPI 3 of clones *P. tomentosa* ‘B331’ and *P. popularis* ‘35–44′. Although the bacterial suspension could diffuse inside the leaves of *P. trichocarpa*, the pressure from the syringe infiltration frequently caused a bump on the infiltration zone, which further led to the separation of the lower epidermis from the rest of the leaf tissue and caused a certain degree of damage to the infiltrated leaves. For clones *P. davidiana* × *bolleana* and *P. alba var. pyramidalis*, the bacterial suspension penetrated the leaf tissue easily, and the infiltrated area was enlarged as the liquid diffused inside the leaf tissue. Notably, the clone *P. davidiana* × *bolleana* was the easiest to work with, and the bacterial suspension spread lightly in all the fully-expanded leaves, particularly in the leaves with leaf Plastochron index (LPI) 4 and the leaves below it, reaching all around the leaves within a few operations in most circumstances (Fig. 1a). LPI was used as indicator of the leaf age [39, 40] in this study, and LPI 4 represents the fourth leaf with a length longer than 20 mm from the plant top.

Next, the transformation event was detected through monitoring the reporter protein expression. The signals of green fluorescent protein (GFP) were easily detected in *P. davidiana* × *bolleana* at 3 days post infiltration (dpi) *in planta* by a Fluorescence Excitation Flashlight (Night Sea, USA) (data not shown). Since the damaged leaf tissue from the mechanical pressure of the syringe tip can emit false fluorescent signals under the Fluorescence Excitation Flashlight, which can lead to great difficulty in evaluating the GFP reporter expression in clones in which the bacterial suspension could not spread well within the leaves, we detected GUS reporter expression at 5 dpi to monitor the differential transformation among the tested clones (Fig. 1b). In clone *P. davidiana* × *bolleana*, the transformation occurred in wide areas in all of the infiltrated leaves. For clone *P. alba var. pyramidalis* (i.e., *P. bolleana*), positive GUS staining was observed in the regions where the agrobacterial suspension had reached, especially in leaf LPI 4. Conversely, no transformation event was observed in the leaves of *P. trichocarpa*, probably due to the poor physiological state of the leaves that had not fully recovered from the infiltration damage. In the other eight clones, GUS activity was detected only near the syringe contact zone of the leaves, consistent with the weak spread of the agrobacterial suspension inside the leaves.

We noticed that easy spreading of the bacterial suspension occurred in the clones *P. davidiana* × *bolleana*, *P. alba var. pyramidalis*, and *P. trichocarpa*. Among them, clone *P. alba var. pyramidalis* is the male parent of the aspen hybrid *P. davidiana* × *bolleana*. This suggests that genetic background might play an important role in the spreadability of the agrobacterial suspension inside the leaf tissue. In order to assess the roles that leaf interior structure might have in the diffusion of the agrobacterial suspension, transverse sections of the leaves of the tested clones were observed (Fig. 1c). Surprisingly, we found that the intercellular air space under the lower side of the leaf was much bigger and more continuous in these three poplar clones, indicating better spread of the agrobacterial suspension inside the leaf tissue. In particular, the clone *P. trichocarpa* had the biggest intercellular air space among all of the tested clones, which extended widely and continuously, and occupied over a half of the leaf transverse area. In contrast, in the other clones, the agrobacterial suspension was limited to a smaller region, since the air space was smaller, and it was more compartmented by the vein tissue cells and compacted mesophyll cells. Additionally, there are other distinct features in the leaves of the clone *P. davidiana* × *bolleana* that might contribute to its good performance in agroinfiltration. In particular, the mesophyll cells are arranged randomly and loosely, and almost every cell is surrounded by an air space, and air spaces even exist between the vein tissue cluster and the lower epidermis (Fig. 1c). These data suggested that the distinct leaf interior structure helped the agrobacterial suspension to spread further and make more contact with leaf cells in *P. davidiana* × *bolleana* than in the other clones, which contributed greatly to its high transient expression efficiency in agroinfiltration.

Since the aspen hybrid *P. davidiana* × *bolleana* was most easily infiltrated (Fig. 1a) and showed relatively high transient expression efficiency (Fig. 1b), which was further verified by quantitative analysis of the enzymatic activity of reporter luciferase (LUC) in those three suspension-spreadable clones *P. davidiana* × *bolleana*, *P. alba var. pyramidalis*, and *P. trichocarpa* (Fig. S1), it was chosen for syringe agroinfiltration in poplar and used in the subsequent experiments.

### Effect of the physiological state of the organism, *Agrobacterium* strains, and chemical components on transient expression efficiency

From the hundreds of infiltrations conducted in this study, we noted large differences in the level of transient gene expression in plants of different physiological states and of different developmental stages, as well as in leaves of different ages. Additionally, the *A. tumefaciens* strain used for infiltration affected the level of transient gene expression dramatically. From the initial experiments, we found that poplar plants cultured on Murashige & Skoog (MS) medium in a growth chamber, which had thinner leaf blades compared with the plants grown in soil, were recalcitrant to agrobacterial syringe infiltration, as the bacterial suspension failed to diffuse inside the leaf tissue. For the plants grown in soil, the lower and older leaves were found to be infiltrated more easily, with the bacterial suspension spreading widely in these tissues. We also found that *A. tumefaciens* strain GV3101 exhibited higher transient expression efficiency than EHA105 through investigating the GFP-Flag reporter expression by immunoblotting at 5 dpi (Fig. S2a, Fig. S8). Furthermore, when the infiltration medium contained 1.6 mM AS, the highest LUC activity was obtained (Fig. S2b). On the basis of the results of the preliminary experiments, the experimental conditions that were reported to influence the transient transformation efficiency of agroinfiltration [16, 18, 29] were further optimized, including the developmental stages of the plant and leaf age, strains of *Agrobacterium*, phase of bacterial growth and bacterial density, concentration of AS, infiltration medium, and duration of expression by quantifying the reporter LUC enzymatic activity.

**Fig. 2:**
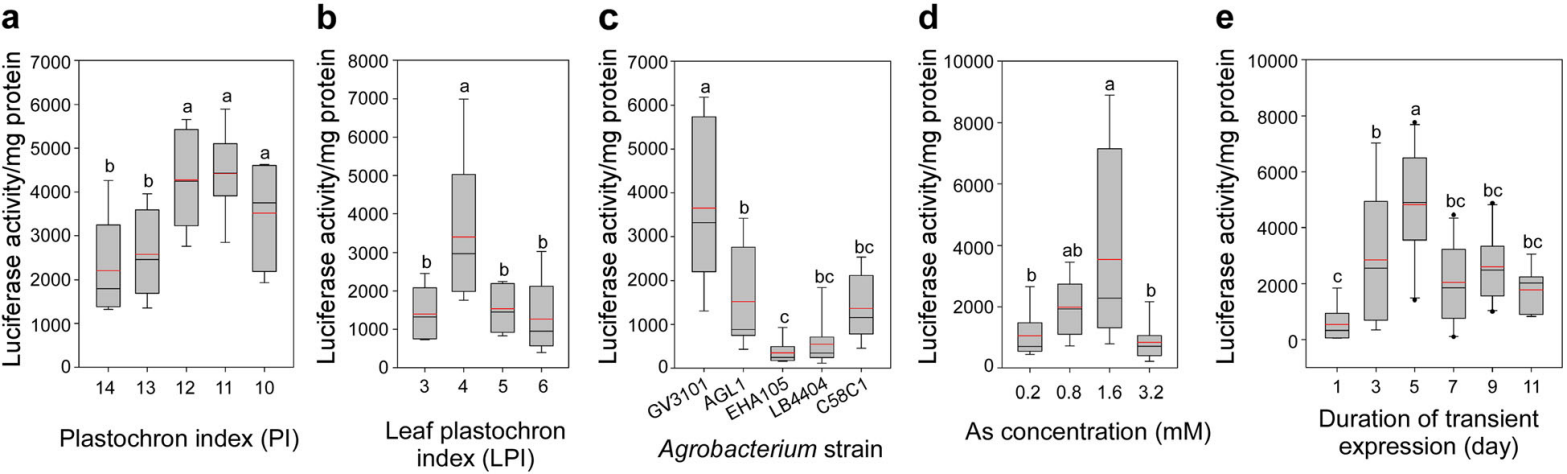
Factors affecting the transient expression efficiency in poplar leaves. Optimization of the experimental parameters was carried out on *P. davidiana* × *bolleana*. **a** Effect of the plant development stage on transient expression efficiency. Syringe agroinfiltration was conducted on leaves LPI 4 using *A. tumefaciens* GV3101 suspended in modified infiltration medium [10 mM MgCl_2_, 5 mM MES-KOH (pH 5.6) and 1.6 mM AS] and evaluated at 5 dpi. **b** Effect of leaf age on transient expression efficiency. The syringe agroinfiltration was conducted on plants PI 11–12 using GV3101 suspended in modified infiltration medium [10 mM MgCl_2_, 5 mM MES-KOH (pH 5.6) and 1.6 mM AS] and evaluated at 5 dpi. **c** Effect of *Agrobacterium* strains on transient expression efficiency. The syringe agroinfiltration was conducted on the leaves LPI 4 of plants PI 11–12 by using the indicated *A. tumefaciens* strains suspended in modified infiltration medium [10 mM MgCl_2_, 5 mM MES-KOH (pH 5.6) and 1.6 mM AS] and evaluated at 5 dpi. **d** Effect of acetosyringone concentration on transient expression efficiency. The syringe agroinfiltration was carried out on leaves LPI 4 of plants PI 11–12 by using GV3101 suspended in the infiltration media [10 mM MgCl_2_, 5 mM MES-KOH (pH 5.6)] supplemented with different concentrations of AS and evaluated at 5 dpi. **e** Effect of the duration of transient expression on the transient expression efficiency. The syringe agroinfiltration was carried on leaves LPI 4 of plants PI 11–12 by using GV3101 suspended in modified infiltration medium [10 mM MgCl_2_, 5 mM MES-KOH (pH 5.6) and 1.6 mM AS]. LUC activity was evaluated at 1, 3, 5, 7, 9, and 11 dpi. The different letters above the bar indicate statistically significant differences, while the same letter indicates no significant difference according to Duncan’s (D) test (*P* < 0.05). The red line shows the average LUC activity (*n* = 8)

Using *A. tumefaciens* strain GV3101, we performed infiltration in leaves LPI 4 from plants at different PI developmental stages of 10, 11, 12, 13, and 14 in order to quantify the effect of plant age on transient gene expression. PI was used as an indicator of plant age [39, 40] in this study, which used the total number of leaves whose lamina length exceeded 20 mm in plants to present the plant age. We found that LUC activity in the younger poplar plants with PI values ranging from 10 to 12 was about twofold higher than that in older plants with PI values of 13–14 (Fig. 2a). Since plants at age of PI 12 exhibited the highest transient expression among the plants of different ages, they were chosen to test the effect of leaf development stage on transient expression efficiency. These tests were also carried out using *A. tumefaciens* strain GV3101. Among the leaves of LPI ranging from 3 to 6, leaf LPI 4 exhibited the highest levels of expression efficiency (Fig. 2b). Thus, in the following experiments, transient assays were performed on leaf LPI 4 in plants PI 12, which typically had been growing for about 2 weeks in soil in the climate chamber.

Next, we assessed the effect of *Agrobacterium* strains GV3101, EHA105, AGL1, LBA4404, and C58C1 on transient assay efficiency by quantifying the reporter LUC enzymatic activity. Significant differences in transient expression efficiency were observed among the strains. Notably, infiltration with the GV3101 strain resulted in the highest LUC activity, whereas strains EHA105 and LBA4404 exhibited the lowest activity (Fig. 2c). We then investigated whether the cell concentration and growth stage of the GV3101 culture influenced the gene expression efficiency. No significant difference was observed among cultures of different bacterial densities and growth stages (Fig. S3a). Nevertheless, the renewed culture showed slightly higher LUC activity than the non-renewed culture (overnight culture) (Fig. S3a). Additionally, the effect of AS dosage on the level of transient gene expression was evaluated using infiltration media [10 mM MgCl_2_, 5 mM MES-KOH (pH 5.6)] containing 0.2, 0.8, 1.6, or 3.2 mM AS in plants PI 12. Again, we found that the highest LUC activity was obtained with 1.6 mM AS, which was about three times higher than that obtained from the medium with 0.2 mM AS (Fig. 2d). Furthermore, we showed that the LUC activity gradually increased after infiltration, reached its maximum at 5 dpi, and decreased slowly thereafter (Fig. 2e). To further improve the transient assay, two types of infiltration media [10 mM MgCl_2_, 5 mM MES-KOH (pH 5.6), and 1.6 mM AS] and [0.5 × MS medium, 5 mM MES-KOH (pH 5.6) and 1.6 mM AS], modified according to reported [29], were compared, which gave very similar results in our experiments (Fig. S3b).

**Fig. 3:**
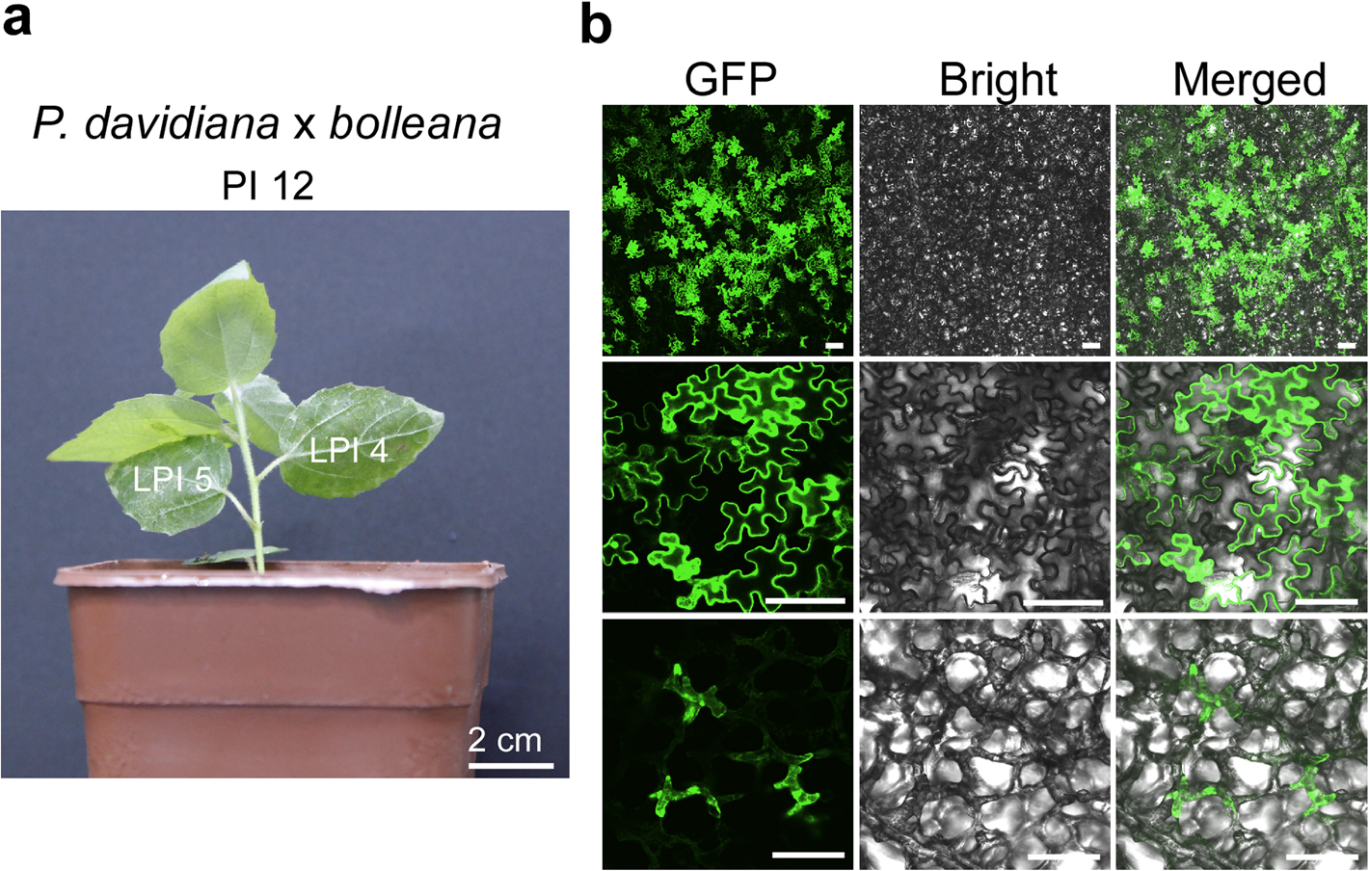
The ideal *P. davidiana* ×*bolleana* plant for agroinfiltration and transient expression of reporter gene. **a** The ideal plant with an age of PI 12 and its optimum leaves of LPI 4 and LPI 5 used for agroinfiltration. The syringe agroinfiltration was conducted using *A. tumefaciens* GV3101 suspended in modified infiltration medium [10 mM MgCl_2_, 5 mM MES-KOH (pH 5.6) and 1.6 mM AS]. **b** Transient expression of green fluorescent protein (GFP) reporter in epidermal cells and mesophyll cells. The expression of GFP was evaluated at 5 dpi. To observe the fluorescent signals in the mesophyll cells, the lower epidermis was removed with a tweezer. The upper two rows show GFP expression in the epidermal cells with different magnitudes of enlargement. The third row shows GFP expression in the mesophyll cells

Summarizing the results from the optimization experiments, the highest transient expression in this study was obtained at 5 dpi when *P. davidiana* × *bolleana* leaves LPI 4 from the plant PI 11–12 were infiltrated with *A. tumefaciens* strain GV3101 cells suspended in the infiltration media [0.5 × MS medium, 5 mM MES-KOH (pH 5.6) and 1.6 mM AS]. The ideal plant and leaves used to achieve high transient expression are shown in Fig. 3a. Additionally, the expression of reporter GFP obtained under the optimal transient transformation conditions is shown in Fig. 3b. The fluorescent signals were detected in both epidermal cells and mesophyll cells. The subsequent experiments were conducted under these optimal transient transformation conditions.

### Subcellular localization of various poplar proteins

**Fig. 4:**
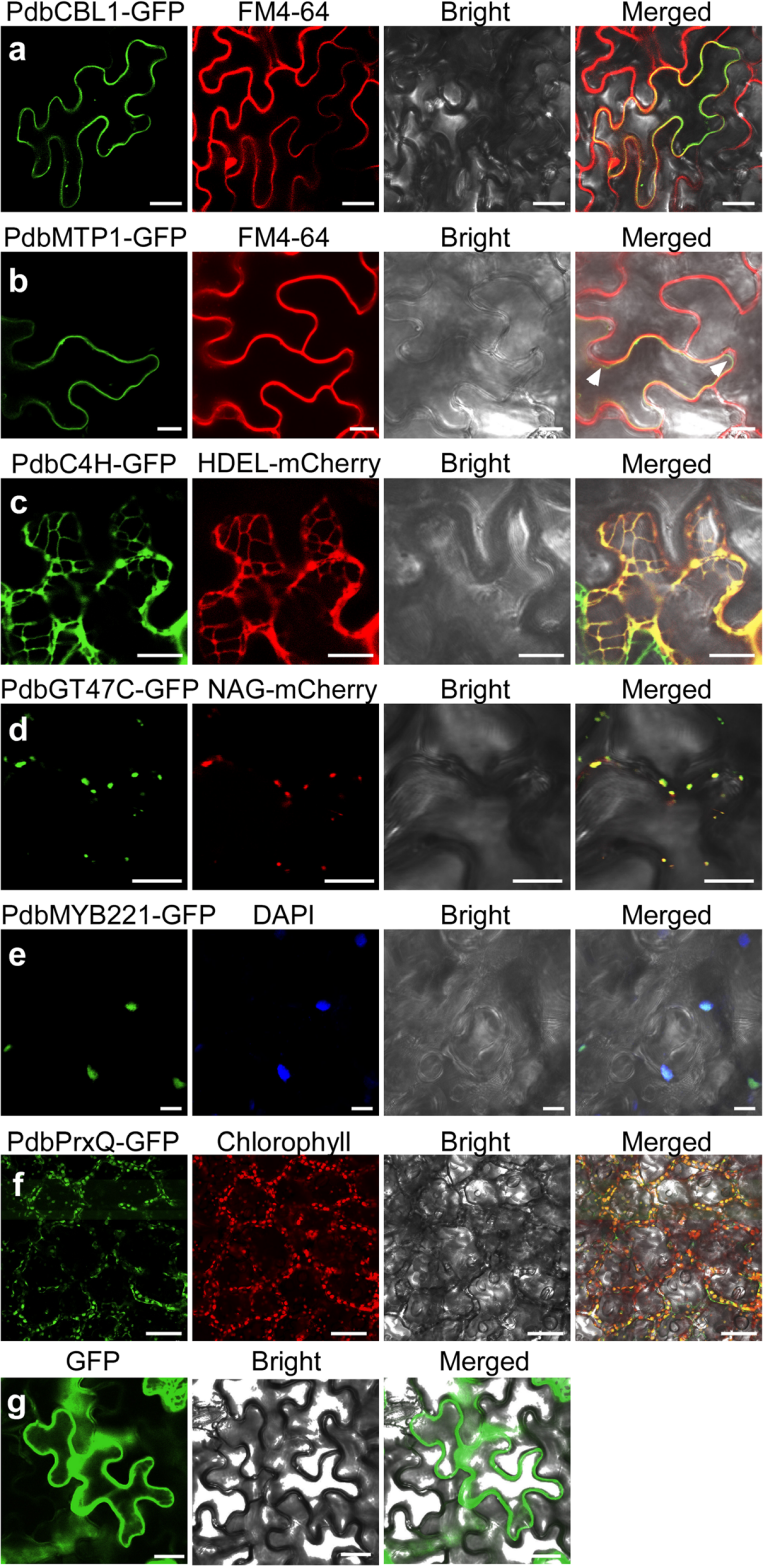
Subcellular localization of various poplar proteins. The various GFP-fused poplar proteins were driven by the Super promoter. The *A. tumefaciens* GV3101 suspension was infiltrated into the leaves of *P. davidiana* × *bolleana* plants under the optimal experimental parameters described above in the Results section. At 5 dpi, the infiltrated leaves were detached, and GFP fluorescent signals were observed under a Nikon inverted fluorescence microscope TE2000-E with excitation at 488 nm and emission at 510 nm. **a** PdbCBL1-GFP localized in the plasma membrane, consistent with FM4–64 staining. **b** PdbMTP1-GFP localized in the tonoplast, distinguished from the FM4–64-stained plasma membrane indicated by white arrows. **c** Colocalization of PdbC4H-GFP in the endoplasmic reticulum (ER) with ER marker HDEL-mCherry. **d** Colocalization of PdbGT47C-GFP in the Golgi with Golgi marker NAG-mCherry. **e** Localization of PtoMYB221-GFP within the nucleus, consistent with DAPI staining. **f** Localization of PdbPrxQ-GFP within plastids, consistent with chlorophyll autofluorescence. **g** Localization of GFP driven under the Super promoter in the cytoplasm and the nucleus

One of the main applications of transient transformation is to monitor the localization of target proteins to subcellular compartments in living cells. In order to validate the transient transformation assay for the subcellular localization of poplar proteins, several types of proteins, targeted to various subcellular compartments, such as the plasma membrane (PM), endomembrane compartments (for example, vacuolar membrane, ER, and Golgi), nucleus, and plastid organelle, were fused with the GFP and expressed via syringe agroinfiltration in poplar leaves *in planta*. We first determined the subcellular localization of PdbCBL1 (Calcineurin B-like Calcium Sensor Protein 1) [41], which localized to the PM and regulated Na^+^/K^+^ homeostasis in *P. euphratica*. PdbCBL1-GFP fusion proteins were localized to the PM in cells, completely overlapping with the fluorescence of PM dye FM4–64 (Invitrogen) (Fig. 4a). Next, localization in endomembrane compartments of several characterized poplar proteins, metal-tolerance protein 1 (MTP1) [42], cinnamate-4-hydroxylase (C4H) [43], and glycosyltransferase family 47 (GT47C) [44, 45] was demonstrated in our transient expression assay. The green fluorescence of PdbMTP1-GFP was visible within the tonoplast, distinguished from PM marked by FM4–64 (Fig. 4b), in accordance with the function of the PtoMTP1 protein for zinc sequestration as a vacuolar zinc transporter at the vacuolar membrane [42]. The fluorescence signals of PdbC4H-GFP were distributed in the reticulate net and overlapped with the red fluorescence of the ER marker protein HDEL-mCherry [46] (Fig. 4c). PdbGT47C displayed a punctate pattern and overlapped with the co-expressed Golgi vesicle marker NAG-mCherry, consistent with its function in the biosynthesis of glucuronoxylan during secondary wall formation [44, 45] (Fig. 4d). Moreover, PtoMYB221, a member of the MYB transcription factor for the regulation of lignin biosynthesis [47], was shown to localize in the nucleus, confirmed by co-localization with the DAPI (Sigma)-stained nucleus (Fig. 4e). PdbPrxQ, which is a member of the peroxiredoxin family involved in detoxifying peroxides [48], was defined in the chloroplasts and co-localized with chlorophyll auto-fluorescence (Fig. 4f). In comparison, the control GFP signal driven under the Super promoter was found to express universally in the cytoplasm and the nucleus of the epidermal cells (Fig. 4g). Hence, the expression of multiple target proteins in the transformed cells validated the transient transformation assay and its practicality for the subcellular localization of *Populu*s genes in a homologous plant system.

### Co-transformation for protein–protein and protein–DNA interactions

**Fig. 5:**
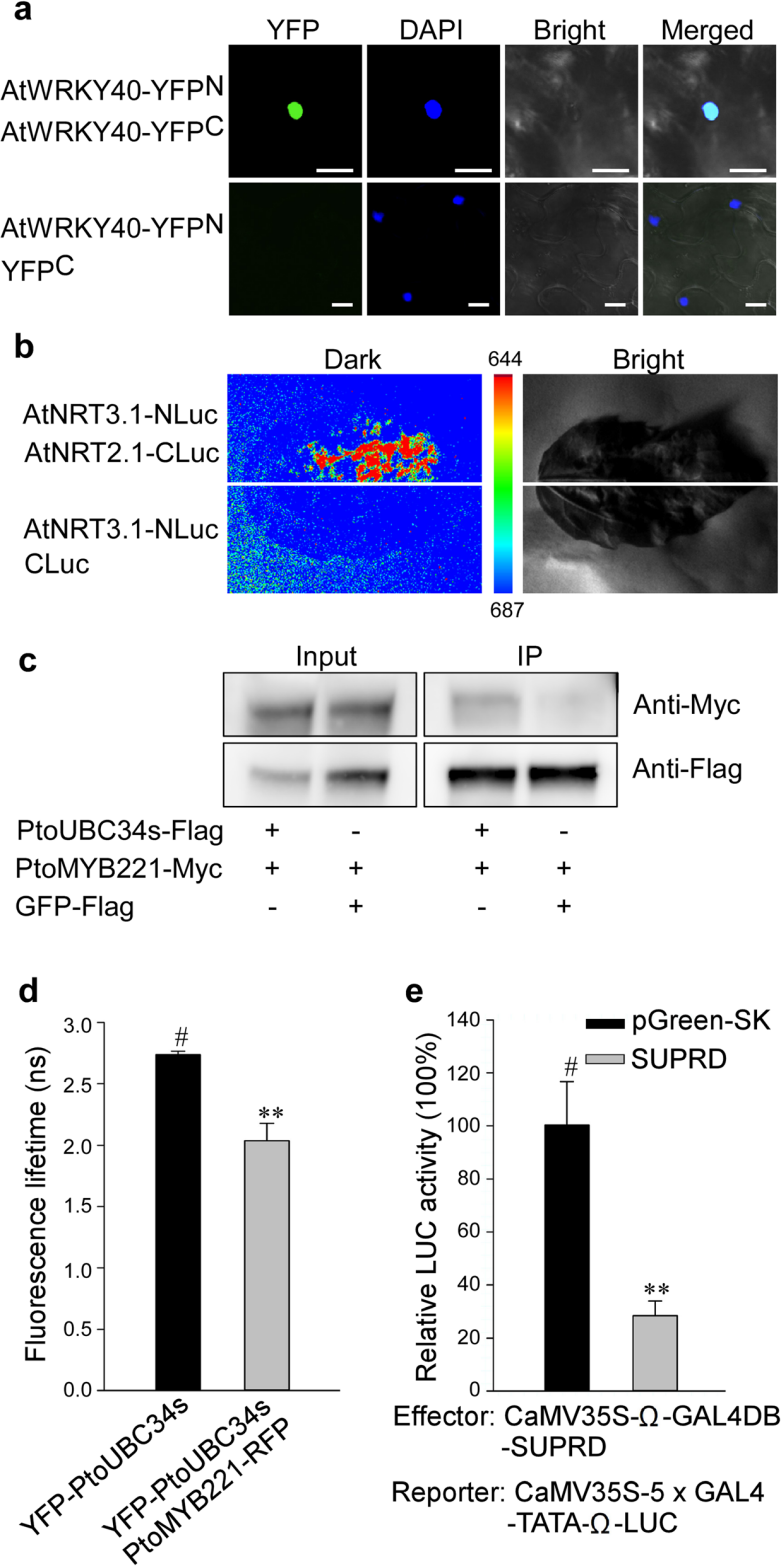
Protein–protein and protein–DNA interactions in the cells of the poplar leaves. Protein–protein interactions were illustrated with various methods using transient co-transformation in the poplar leaves by syringe infiltration under the optimal experimental parameters described above in the Results section. **a.** BiFC assay of AtWRKY40-YFP^N^ and AtWRKY40-YFP^C^. The combination of AtWRKY40-YFP^N^ and YFP^C^ was used as a negative control. At 5 dpi, the infiltrated leaves were detached, and the YFP fluorescent signals were observed under a Nikon inverted fluorescence microscope TE2000-E. YFP signals and DAPI fluorescence overlapped in the nucleus. **b.** Split luciferase assay of AtNRT3.1-Nluc and AtNRT2.1-Cluc, showing stronger LUC activity compared to the negative control combination of AtNRT3.1-Nluc and Cluc. At 5 dpi, the transformed leaves were infiltrated with 2 mM luciferin by using a syringe without a needle, left in dark for 6 min to quench the fluorescence, and then detached for the luminescence intensity assay. The color scale shows the luminescence intensity, with blue indicating the lowest and red the highest. **c.** Images of the co-immunoprecipitation assay show the interaction of PtoUBC34s with PtoMYB221. PtoUBC34s-Flag was co-expressed with PtoMYB221-Myc, and protein extracts were incubated with anti-Flag coupled agarose. Immunoprecipitates (IP) and input proteins were analyzed by immunoblotting using anti-Flag and anti-Myc antibodies. The uncropped images can be found in Fig. S7. **d.** In vivo Förster resonance energy transfer (FRET-FLIM) measurement of co-expressing YFP-PtoUBC34s and PtoMYB221-RFP, with YFP-PtoUBC34s as the donor. The YFP-PtoUBC34s donor alone used as a negative control. The data are presented as means ±SE (*n* = 3). *P < 0.05, Student’s *t*-test. **e.** Dual LUC assay detected the repression ability of the EAR-like motif repression domain of SUPERMAN (SUPRD) [48, 54]. Relative LUC activities were measured after co-transformation with the reporter and effectors, where pGreen-SK was used as a control vector. The data are presented as means ±SE (*n* = 6). ** *P* < 0.01, Student’s *t*-test

Another standard application of a transient transformation assay is to detect protein–protein and protein–DNA interactions in living cells. The commonly used methods for these studies, such as BiFC (bimolecular fluorescence complementation), split-luciferase, CoIP (co-immunoprecipitation), FRET-FLIM (Förster resonance energy transfer) [49], and transaction assay, were performed here by syringe agroinfiltration in poplar. First, a BiFC assay was verified by the interaction of AtWRKY40 with itself due to the formation of homodimers [50]. The YFP signal was detected in the nucleus after co-expression of AtWRKY40-YFP^N^ with AtWRKY40-YFP^C^, which was verified by DAPI staining, whereas no fluorescence was observed in the negative control combinations, indicating the interaction of AtWRKY40 with itself (Fig. 5a). Then, a split-luciferase assay was conducted with the nitrate transporters AtNRT2.1 and AtNRT3.1, which formed a complex in the PM [51, 52]. The co-expression of AtNRT3.1-Nluc and AtNRT2.1-Cluc led to stronger LUC activity in the poplar leaves compared to the negative control, which showed only background level LUC activity (Fig. 5b). Next, we selected the protein combination of PtoUBC34 (ubiquitin-conjugating enzyme 34) and PtoMYB221, which formed a complex in the ER [47], to demonstrate a more authentic protein interaction by biochemical co-immunoprecipitation (CoIP) and Förster resonance energy transfer (FRET-FLIM) assay. CoIP showed that Flag-tagged PtoUBC34s (truncated UBC34) co-precipitated with Myc-tagged PtoMYB221 (Fig. 5c), suggesting the formation of a complex. The FRET-FLIM experiment showed that the lifetime of the PtoUBC34s-YFP fluorescent signal was significantly reduced when it was co-expressed with PtoMYB221-RFP (Fig. 5d), implying them being close enough on a nanometer scale and the formation of a complex. Lastly, we demonstrated the transcriptional repression activity of SUPRD [47, 53] with a significant reduction of relative LUC activity when co-expressing effector GAL4BD-SUPRD with reporter *35S*:*GAL4*-*LUC* (Fig. 5e). This transient assay was able to demonstrate *Populus* protein interaction *in planta* by BiFC, Split-luciferase, CoIP, and FRET-FLIM, as well as conduct the transaction assay.

### Generation of stably transformed plants from transiently transformed leaves

To determine the possibility of integration of the transient transferred gene into the plant genome, we investigated whether stably transformed plants could be regenerated from the transiently transformed leaves. Two transformation vectors expressing GFP with hygromycin resistance or expressing GUS with kanamycin resistance were used. These reporter genes allowed us to use the GFP fluorescence or GUS staining as a marker to follow the various steps of plant regeneration. Via direct organogenesis, all of the regenerated plants were non-transgenic in this study. Conversely, most of the regenerated shoots that developed via the callus-induced indirect organogenesis process were confirmed to be transformed (Table 1, Fig. S4). Notably, about 67–75% of positive calli were able to produce at least one transgenic plant. As many as about 54–97% of explants formed positive calli with shoot and leaf primordia, and 41–67% of explants regenerated at least one transgenic plant (Table 1). In brief, the *Agrobacterium* syringe-infiltrated poplar leaves could be used to generate stably transformed plants with high efficiency.

**Table 1:** Generation of stably transformed plants from transiently transformed leaves

	Number of explantsa	Explants forming positive calli (%)^**b**^	Positive calli producing transgenic plants (%)^**c**^	Transformation frequency (%)^**d**^	No. of plants
*Super*:*GFP*	37	54.1	75.0	40.5	15
*35S*:*GUS*	23	95.7	75.0	65.2	15
*35S*:*GUS*	30	96.6	66.7	66.6	20
**Total**	90	–	–	–	50

^a^ The sum of explants;

^b^ The percentage of explants that formed positive calli with shoot and leaf primordia;

^c^ The percentage of positive calli that produced at least one transgenic rooted shoot;

^d^ The percentage of explants that regenerated at least one transgenic rooted shoot

### Induction of xylem vessel element differentiation and secondary wall deposition in the leaves of poplar

**Fig. 6:**
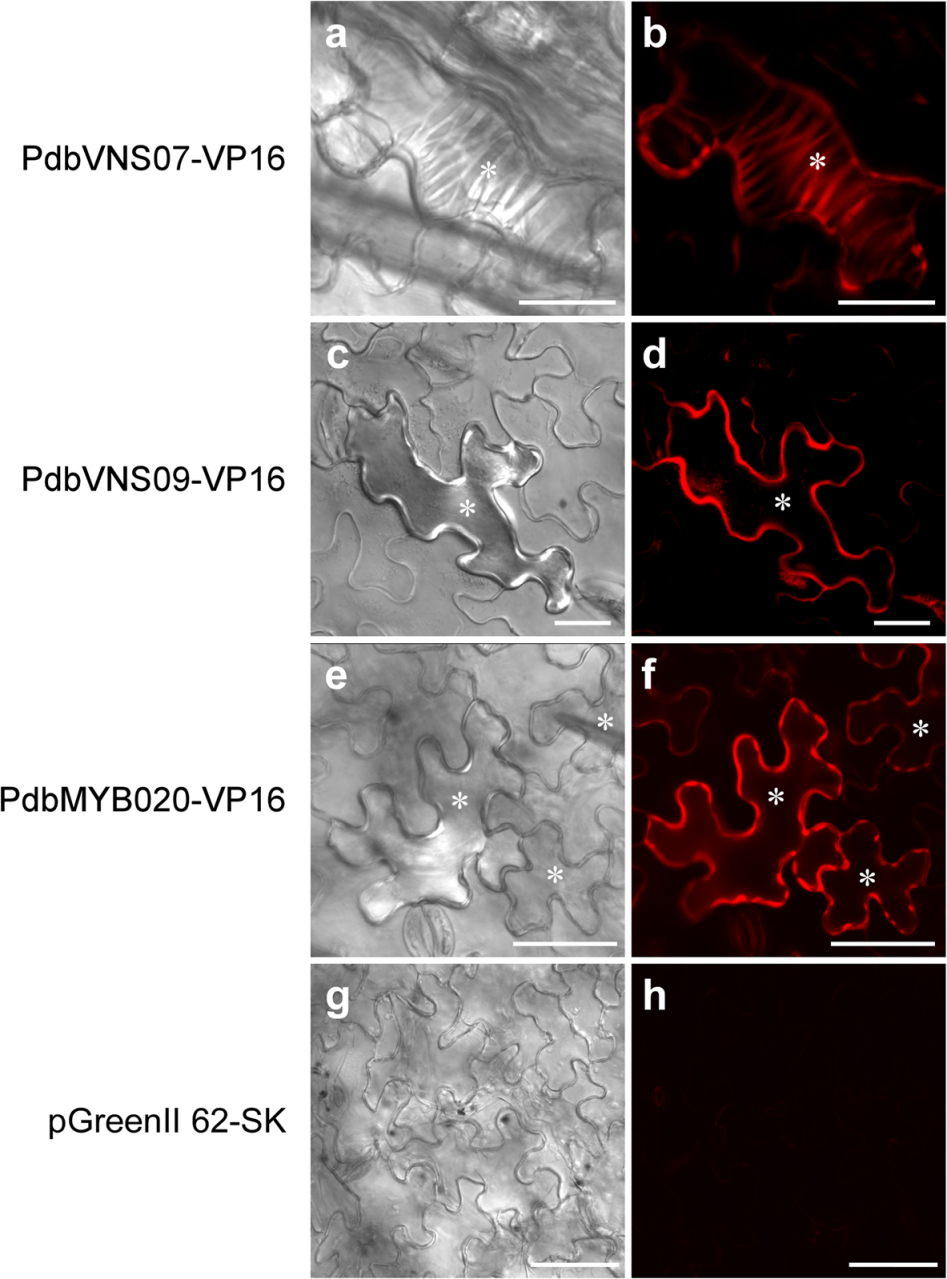
Induction of protoxylem tracheary element differentiation and secondary wall deposition in the epidermal cells. Transient overexpression of PdbVNS07 (VND-, NST/SND- and SMB-related protein), PdbVNS09 or PdbMYB020 fused with the activation domain of the herpes virus VP16 protein in poplar leaves via syringe agroinfiltration resulted in ectopic secondary wall deposition in the epidermal cells. Transiently transformed leaves were detached, stained with basic fuchsin at 10 dpi, and observed with a confocal microscope for secondary walls. **a** and **b.** Epidermal cells overexpressing PdbVNS07-VP16, showing transdifferentiation of protoxylem-like vessel elements with annular and spiral thickenings in the cell wall. **c** and **d.** Epidermal cells overexpressing PdbVNS09-VP16, showing the ectopic secondary wall deposition. **e** and **f.** Epidermal cells overexpressing PdbMYB020-VP16, showing band-like secondary wall thickening. **g** and **h.** Epidermal cells overexpressing the control vector pGreenII 62-SK, showing no secondary wall thickening. **a**, **c**, **e**, and **g** are images of differential interference contrast; **b**, **d**, **f**, and **h** are images of the basic fuchsin stain. White stars indicated ectopic secondary wall deposition

To further explore the potential of this transient expression system for characterizing genes involved in SCW formation, we transiently overexpressed three key activators of SCW biosynthesis, namely, PdbVNS07/WND6A (VND-, NST/SND- and SMB-related proteins, also called WND), PdbVNS09/WND2A, and PdbMYB020, in poplar leaves and succeeded in activating secondary wall biosynthesis in epidermal cells in a specialized manner for each gene (Fig. 6). The overexpression of PdbNVS07/WND6A, a member of the VND group [54], resulted in transdifferentiation of the epidermal cells into protoxylem-like vessel elements with annular and spiral thickenings in the cell wall (Figs. 6a–b), functioning similarly to its *Arabidopsis* ortholog VND7 as a master regulator of plant protoxylem vessel element formation [55, 56]. Leaf epidermis overexpressing PdbVNS09/WND2A showed obvious and massive ectopic secondary wall thickening (Figs. 6c–d), which is consistent with its key role as one of the master switches of SCW biosynthesis in fiber cells [54, 57, 58]. PdbMYB020, another level of key switches of secondary wall biosynthesis and functioning downstream from VNSs master switches [54, 59]**,** resulted in band-like secondary wall thickening in epidermal cells (Figs. 6e–f). Therefore, xylem vessel element differentiation and secondary wall deposition could be induced in vivo in the epidermal cells of poplar leaves through our transient transformation method.

## Discussion

Agroinfiltration has been widely used for high-throughput gene functional studies in many species due to its simplicity, speed, and efficiency [16, 31–35, 60]. Although *Agrobacterium* vacuum infiltration had been established in hybrid aspen *P. tremula* × *tremuloides* [29], the complex operation and typically weak expression have limited its usefulness in functional genomics research in poplar. In this study, we enhanced the *Agrobacterium* syringe infiltration method using the aspen hybrid clone, *P. davidiana* × *bolleana*. Throughout the optimization of the key experimental conditions, this clone exhibited a high level of transient expression and was as easy to work with as the more widely used *N. benthamiana*. The high transformation efficiency enabled subcellular localization of the *Populus* proteins, allowing protein–protein interactions and transcriptional regulation analysis to be fulfilled in a homologous plant system. Furthermore, this method provided an effective alternative to stable genetic transformation as well as a new approach for characterizing the genes involved in secondary wall formation in poplar.

The aspen hybrid clone *P. davidiana* × *bolleana*, also called Shanxin Yang in Chinese, is widely grown in the northern part of China. It was selected as the preferential clone for *Agrobacterium* syringe infiltration since it was the most easily infiltrated and showed relatively high transient expression efficiency. In this clone, the bacterial suspension spread easily through the leaf lamina, which was previously reported to be a key factor for high-level transient expression in agroinfiltration because it maximizes the physical access of the agrobacteria to leaf cells [12, 17, 61]. This speculation is supported by data in *Arabidopsis* [16], grapevine [35], and potato [33], in which the highest transformation efficiency is consistent with the amenability of the agrobacterial suspension to diffuse inside the leaf tissue, as demonstrated in the grapevine cultivar ‘Aleatico’ and potato cultivar ‘Katahdin’. Through investigating the inner structure of the leaves of all the tested poplar clones, we found that the spreadability of the agrobacterial suspension was associated with the volume of the intercellular air spaces within the leaves, which facilitated agrobacterial spread inside the leaf, and sometimes over the vein networks, since the air there was easily replaced with the agrobacterial suspension with gentle pressure on the lower side of the leaf. Good spreadability typically resulted in a high level of transient expression, as demonstrated in clones *P. davidiana* × *bolleana* and *P. alba var. pyramidalis* (Fig. 1 and Fig. S1). However, there was an exception in that the clone *P. trichocarpa* showed the largest intercellular air space and good spreadability, but a very low level of transient expression (Fig. 1 and Fig. S1). We proposed that, compared with the other clones, the vast and continuous air spaces made the leaves of *P. trichocarpa* more likely to be damaged severely during agroinfiltration. With the vast intercellular air space, the weight of the large amount of bacterial suspension within the leaves caused separation of the lower epidermis from the rest of the leaf tissue, as described in the Results section. As a result of the poor physiological state of the leaves, transformation of the leaf cells often failed despite the wide spread of the agrobacterial suspension inside the leaves (Fig. 1 and Fig. S1). In addition to the volume of the intercellular air space within the leaves, we found that the transient transformation efficiency of agroinfiltration was also affected by the arrangement of the mesophyll cells and leaf vein networks (Fig. 1). In the clone *P. davidiana* × *bolleana*, the loosely arranged mesophyll cells afforded them a better chance to make contact with the agrobacterial cells and then be transformed. On the contrary, the relatively smaller and compartmented intercellular space, and the compacted mesophyll cells, restricted the spread of the infiltrated suspension and transformation of the leaf cells in clones *P. alba* × *glandulosa* ‘84 K’, *P. tomentosa* ‘741’, and *P. euramericana* ‘74/76’. Additionally, the restriction of the agrobacterial suspension by leaf vein networks has also been demonstrated in lettuce and tomato [16]. These data demonstrate that the amenability of a plant to syringe agroinfiltration is associated with the interior structure of the leaves.

Interestingly, many of the factors reported to be important for vacuum agroinfiltration in hybrid aspen *P. tremula* × *tremuloides*, such as bacterial density, growth stages, and infiltration medium [29], did not have obvious effects in aspen hybrid *P. davidiana* × *bolleana* under our conditions (Fig. S3). In this study, we found that the physiological condition of the plants played essential roles in efficient syringe agroinfiltration in *P. davidiana* × *bolleana*. Specifically, we learned that the young aspen hybrid plants, which underwent 3 weeks of growth on MS medium in a growth chamber and then 2 weeks of growth in soil in a climate chamber, reaching an approximate plant age of PI 12, exhibited the highest levels of expression efficiency (Fig. 2a). Among leaves of different ages in plant PI 12, LPI 4 was the easiest to infiltrate and showed the highest expression level (Fig. 2b). There are two possible explanations for this result. First, the good performance of leaf LPI 4 from plant PI 12 (Figs. 2a–b) was attributed to its specialized physiological state. This leaf was normally initiated and had grown to less than 1 cm in length on MS medium under the growth chamber conditions and developed more rapidly in the soil under climate chamber conditions, and had fully expanded by the time of infiltration. Further, its vigorous cells that recently experienced rapid cell expansion facilitated a high level of transient transformation, as previously suggested [16, 33]. Second, this leaf was found to have less pubescence compared to the leaves that developed later in the climate chamber, which further facilitated the syringe infiltration. The variation in pubescence might be the result of the differences in water availability between the growth chamber and the climate chamber, with leaf LPI 4 and the younger leaves undergoing organogenesis in the former and latter, respectively. The effect of water availability on pubescence development was also reported for the desert shrub *Encelia farinose* [62].

In this study, we developed an alternative procedure for effective *Populus* genetic transformation using agroinfiltrated leaves as explants. The integration of the transferred genes by agroinfiltration was also reported in tobacco [31] and grapevine [35]. This procedure increased the transformation frequency of aspen hybrid *P. davidiana* × *bolleana* by up to 41–67% (Table 1), which is much higher than that obtained from the routine genetic transformation procedure where leaf disk explants were co-cultivated with *Agrobacterium* liquid culture and shoots were regenerated via direct organogenesis (16.4% transformation frequency) [63]. The higher transformation frequency in this study was attributed mainly to the effectiveness of early selection for transformants during the callus-induction stage, which has been reported to be beneficial for successful transformation [36, 64]. During the indirect organogenesis process, calli formed on the explant’s cut surface, grew slowly along the medium surface, and made close contact with the selective medium during the callus-induction stage, which allowed the transformed cells to multiply under the selective pressure and then minimized the number of non-transgenic escapes (Fig. S5). On the contrary, in the direct organogenesis process, callus-like tissues were normally initiated on the cut of the midrib and the secondary veins in a leaf, probably from cambium cells inside these major veins, which the agrobacterial cells were not able to reach through syringe agroinfiltration due to the tightly aligned vascular bundle sheath cells around the veins. These callus-like tissues were visible on the upper side of the explant’s cut surface after 10 days of culture and grew rapidly upward from the surface of the leaf lamina, which prevented them from directly contacting the selective medium (Fig. S5). As a result of the ineffective selection, all the regenerated plants via direct organogenesis were confirmed to be non-transgenic in this study. The importance of effective selection for transformants in the early stage of the culture process may be further illustrated by the high frequency of positive calli producing at least one transgenic plant (67–75%) (Table 1). Additionally, the generation of stably transformed plants further verified that *Agrobacterium* syringe infiltration was able to target the heterologous genes in mesophyll cells beyond the epidermal cell, as shown in Fig. 3b, since the epidermal cells were resistant to dedifferentiation and had no potential to form callus and further develop into plants [35]. Although the generation of stably transformed lines normally takes 2–4 months, much longer than that for transient expression, the method set out for generating stably transformed poplar lines in this protocol provides a convenient approach to study genes in cell types other than the leaf epidermis. In this case, after the transient expression analysis of the fluorescent fusion protein was performed in the leaf epidermis, the sterilized infiltrated leaves could be directly used for callus induction and then shoot regeneration, circumventing the routine steps of explant inoculation with *Agrobacterium* and co-cultivation of *Agrobacterium*-mediated poplar transformation.

Furthermore, we showed that the agrobacterial syringe infiltration method could be used for in vivo activation of the specialized processes of SCW biosynthesis in the epidermal cells of poplar leaves by overexpressing master activators of secondary wall formation, PdbVNS07/WND6A, PdbVNS09/WND2A, and PdbMYB020 (Fig. 6), in which the activation activity of these key regulators was enhanced through fusion with the activation domain of the herpes virus VP16 protein, as reported in *Arabidopsis* [65]. The overexpression of PdbNVS07/WND6A induced transdifferentiation of the epidermal cells into protoxylem-like vessel elements (Figs. 6a–b), and PdbVNS09/WND2A and PdbMYB020 resulted in ectopic secondary wall deposition in the epidermal cells (Figs. 6c–f). Since vascular tissue is deeply embedded in the plant, it is difficult to analyze the process of vessel element development in detail. For this reason, the in vitro induction system of xylem vessel elements from *Zinnia* suspension cells [66], *Arabidopsis* suspension cells [55], and *Arabidopsis* excised cotyledons [67] was established with effort and has provided fundamental information on xylem vessel element development. Thus, the success in inducing secondary wall formation in poplar leaves provides a powerful tool for dissecting the molecular mechanisms regulating vascular development in poplar. For example, the comprehensive gene expression profile analysis in those SCW-producing epidermal cells will contribute to elucidating the specialized regulatory mechanism of SCW formation and vessel element differentiation of woody plants in a high-throughput manner in the near future.

## Conclusions

By widely exploring suitable *Populus* clones for syringe infiltration and optimizing the experimental parameters, we developed a syringe agroinfiltration assay in poplar. The highest transient expression in this study was obtained at 5 dpi when *P. davidiana* × *bolleana* leaves LPI 4 from plants PI 11–12 were infiltrated with *A. tumefaciens* strain GV3101 cells suspended in infiltration media containing 1.6 mM AS. The infiltrated leaves in one single plant were sufficient for both RNA and protein analysis. This approach will be useful for the rapid and high-throughput characterization of *Populus* genes, such as analyses of the subcellular localization of gene products and the interaction between proteins and proteins or DNA, the production of stable transformants, and the elucidation of gene biological function and molecular mechanisms, e.g., in the developmental process of protoxylem tracheary elements and the biosynthesis of SCW. Since the transient transformation is conducted in intact plants, this system allows gene function to be elucidated in diverse genetically modified backgrounds, especially in overexpression transgenic lines, RNAi-based gene silencing lines, artificial microRNA-based gene silencing lines, and genome editing lines, either via transient overexpression or silencing of the target genes through syringe agroinfiltration. This makes it possible to manipulate multiple genes in perennial trees, in which crossing between mutant (or transgenic) lines normally takes years.

## Methods

### Plant materials and growth conditions

*Populus* clones *P. davidiana* × *bolleana* (known as Shanxin yang, a gift from Prof. Zhang [63], originated in the Academy of Forest and Environment of Heilongjiang Province, Heilongjiang, China), *P. tremula* × *alba* ‘INRA 717-1B4’ (a gift from Prof. Z. Ye, University of Georgia, Georgia, USA, originated in the French National Research Institute for Agriculture, Food and Environment, France), *P. alba* × *glandulosa* ‘84 K’ (introduced from South Korea by the Chinese Academy of Forestry, Beijing, China), *P. euramericana* ‘74/76’ (introduced from Italy by the Chinese Academy of Forestry, Beijing, China), *P. tomentosa* ‘741’ (Hebei Agricultural University, Hebei, China), *P. tomentosa* ‘B331’ (Beijing Forestry University, Beijing, China), *P. tomentosa* ‘BJHR01’ (cooperatively bred at the Beijing Academy of Agriculture and Forestry Sciences, Beijing, China, and Beijing Forestry University, Beijing, China), *P. popularis* ‘35–44’ (Chinese Academy of Forestry, Beijing, China), *P. davidiana* (a gift from Prof. T. Jiang, Northeast Forestry University, Heilongjiang, China, originated in Heilongjiang, China), *P. alba var. pyramidalis* (a gift from Prof. C. Xu, Southwest University, Chongqing, China, originated in Xinjiang, China), *P. trichocarpa* (Nisqually-1, North America) were initially cultured on Murashige & Skoog (MS) medium (Phytotech, M519) supplemented with 3% sucrose and 0.6% agar. Clonal propagation of the poplar plants was conducted as described by Wang et al. [68], and plants were cultured on MS sterile medium under a 16 h/8 h day/night photoperiod at 25 °C in a growth chamber. After 3 weeks of growth, the rooted plantlets were transferred into soil and grown under a 16 h/8 h day/night photoperiod at 25 °C in a climate chamber. Once transferred into the soil, the plants were covered with transparent lids for one week to prevent excess water loss from the leaves. For the initial experiments, in which poplar clones were screened, poplar plants were grown in a climate chamber for one month before infiltration. For the experiment assessing the effect of plant age and leaf age of *P. davidiana* × *bolleana* on transient expression efficiency, five batches of 3-week-old in-vitro cultured plantlets were transferred to soil at an interval of 3 days. When the first batch of plants in the soil reached the age of PI 14 after 3–4 weeks of growth, agroinfiltration was performed on LPI 4 leaves [39, 40] of different developmental stage plants of PI 10, 11, 12, 13, 14, and on leaves LPI 3–6 from plants PI 12. For later experiments, to evaluate the effects of AS concentration, bacterial growth phase, bacterial cell density, infiltration medium, and duration of transient expression on the transient expression efficiency, plants were grown in a climate chamber for 2 weeks, i.e., PI ~ 12.

### Leaf syringe infiltration

*Agrobacterium tumefaciens* strain GV3101, *A. tumefaciens* EHA105, and *A. rhizogenes* C58C1 were obtained from the laboratory, and *A. tumefaciens* strains AGL1 and LBA4404 were purchased from Shanghai Weidi Biotechnology Co., Ltd. (Shanghai, China). The day before infiltration, agrobacteria with a specific binary vector (Fig. S6) starting from a single clone on agar plates were cultured in LB liquid medium overnight at 28 °C on a shaker. A new bacterial culture was started the next morning by inoculating fresh medium with the old suspension cultures (1/50 ratio, v/v) for another 5–6 h until OD_600_ ~ 1. Then, the cultures were transferred to Eppendorf tubes and centrifuged at 8000 *g* for 2 min at room temperature and suspended in the reported infiltration medium [10 mM MgCl_2_, 5 mM MES-KOH (pH 5.6) and 0.2 mM AS] [29] to a final OD_600_ ~ 1 in the initial experiments. For later experiments, the agrobacterial cultures were suspended in the modified infiltration medium [10 mM MgCl_2_, 5 mM MES-KOH (pH 5.6) and 1.6 mM AS] instead. For the experiments evaluating the effect of the growth stages and cell concentrations of the bacterial culture on the transient expression efficiency, either the overnight culture with an OD_600_ of ~ 2.0 was used directly for infiltration after being suspended in the modified infiltration medium with a final OD_600_ of 0.2, 0.5, 1.0, 1.5 or 2.0, or the renewed culture was grown to a final OD_600_ of 0.2, 0.5, 1.0, 1.5 or 2.0, and re-suspended in the modified infiltration medium with the same OD as the original culture. The bacterial suspension was placed in the dark for 1–2 h at room temperature before agroinfiltration.

The plants used for syringe infiltration were described above. The bacterial suspensions were infiltrated into the poplar leaves through the stomata by pressing the tip of a 1-mL syringe without a needle against the lower side of the leaf and applying gentle pressure on the plunger. Normally, multiple injections were applied to a single leaf in order to enlarge the infiltrated parts. The infiltrated area of the leaves was circled by a marker pen, following which the poplar plants were grown in a climate chamber for 5 d and were used for the evaluation of gene expression. For the co-expression of two or more target genes, an equal volume of *A. tumefaciens* suspension with OD_600_ ~ 1.0 in the infiltration medium was mixed before infiltration.

### LUC activity assay

The CaMV *35S*:*LUC* vector (Fig. S6a) was a gift from Prof. Wang (China Agricultural University, Beijing, China) [69]. Total protein was extracted from the infiltrated area of the leaves, and 50 μL of protein extract was used to detect firefly luciferase activity by using Luciferase Assay Reagent (Promega) and a luminescence reader (Glo-Max®20/20; Promega) according to the manufacturer’s protocol. The concentration of the protein was quantified by a Bradford protein assay. The LUC activity was calculated by the light intensity per microgram protein. Each data point represented at least eight replications. Three independent experiments were performed.

### GUS staining

The leaves, callus, and plant that had been transformed with pCAMBIA2301-CaMV *35S*:*GUS*-intro (Fig. S6b) were stained for β-glucuronidase (GUS) activity as described [70]. A plant-derived intron was inserted inside *GUS* to avoid the expression of the reporter gene in the *Agrobacterium*.

### Subcellular localization and BiFC assay

Several proteins of *P. davidiana* × *bolleana* were investigated for their subcellular localization. To prepare GFP-tagged vectors, the coding regions of *CBL1*, *MTP1*, *C4H*, *GT47C*, *MYB221*, and *PrxQ* were amplified by reverse transcription polymerase chain reaction (RT-PCR) using specific primers, in which the corresponding restriction enzymes were included (Table S1), and then cloned into the binary vector pSuper1300-*SUPER*:*GFP* (Fig. S6c). The *A. tumefaciens* GV3101 suspension harboring one of these vectors was infiltrated into the LPI 4 leaves of *P. davidiana* × *bolleana* plants PI 11–12 by using a syringe under the optimal experimental parameters described above in the Results section. At 5 dpi, the infiltrated leaves were detached, and GFP fluorescent signals were observed under a Nikon inverted fluorescence microscope TE2000-E with the excitation wavelength at 488 nm and the emission wavelength at 510 nm. To identify the subcellular compartments, the plasma membrane was stained with FM4–64 (20 mg/L, Invitrogen) for 1 min, the nucleus was stained with DAPI (1 mg/mL, Sigma) for 10–20 min, the ER was indicated using the ER marker fusion protein HDEL-mCherry [71], and the Golgi was indicated using the Golgi marker fusion protein NAG-mCherry [71]. The fluorescence of FM4–64 was detected with excitation at 543 nm and emission at 610 nm, and DAPI with excitation at 358 nm and emission at 461 nm. Red fluorescence signals of fusion proteins HDEL-mCherry and NAG-mCherry were monitored at an excitation wavelength of 543 nm and emission wavelength of 610 nm. Chlorophyll was detected by its auto-fluorescence at an excitation of 488 nm and emission of 681 nm.

For the BiFC assay, the coding region of *AtWRKY40* was amplified using specific primers (Table S1) and cloned into the pSPYNE173 (NE) and pSPYCEM (CE) vectors (Fig. S6e) [72]. Fluorescence signals of YFP were observed at an excitation of 488 nm and emission of 510 nm. Visualization of the nucleus with DAPI dye was conducted as described above.

### Split luciferase assay

GV3101 *Agrobacteria* carrying the constructs of *35S*:*AtNRT3.1*-*Nluc*, *35S*:*AtNRT2.1*-*Cluc*, *35S*:*Nluc*, and *35S*:*Cluc* (Fig. S6f) was gifted from Prof. Wang (China Agricultural University, Beijing, China). A split-luciferase assay was conducted as described [73] with some modifications. Specifically, the transformed leaves were infiltrated with 2 mM luciferin by using a syringe without a needle at 5 dpi and then left in the dark for 6 min to quench the fluorescence. Luminescence intensity was captured by a low-light cooled CCD imaging apparatus (Lumazone PyLoN2048B, Roper Scientific) with an exposure time of 5–10 min when the camera was cooled to − 110 °C. Image acquisition was operated and processed by Light Field software.

### Western blot and co-immunoprecipitation assays

For the western blot, the infiltrated leaves with *A. tumefaciens* GV3101 or EHA105 carrying *Super*:*GFP*-*Flag* (Fig. S6d) were harvested at 5 dpi. The extraction of total proteins from the infiltrated parts of the leaves and western blot assay were performed according to Ticconi et al. [71]. Briefly, 15–30 μg of protein was separated on 10% SDS-PAGE. Anti-Flag (MBL) and anti-Actin (Abmart) were used as the primary antibodies. Image acquisition was captured by a CCD remote control science imaging system (LAS-4000, FUJIFILM). For the co-immunoprecipitation assay, *A. tumefaciens* GV3101 harboring *Super*:*PtoUBC34s*-*Flag* or *Super*: *PtoMYB221*-*Myc* (Fig. S6g) was used for infiltration. The co-immunoprecipitation assay was conducted as described previously [47].

### FRET-FLIM assay

The interaction between PtoMYB221 and PtoUBC34s was described in a previous study [47]. The donor vector pGreen0029-*35S*:*YFP*-*PtoUBC34s* and receptor vector pGreen0029-*35S*:*PtoMYB221-RFP* (Fig. S6h) were co-transferred into poplar leaves via *Agrobacterium*-mediated syringe infiltration as described above. FRET-FLIM was performed on an Olympus inverted FV1200 microscope additionally equipped with a Picoquant picoHarp300 (Germany) controller according to the reported method [74]. The YFP-PdbUBC34s was excited at 488 nm using a picosecond pulsed diode laser operated at a repetition rate of 40 MHz through an objective (40× water immersion, NA 1.2). The emitted light was collected in the same objective and filtered with a 520/35 nm bandpass filter. Fluorescence was then detected by an MPD SPAD detector. The region of interest in the images was selected and acquired with acquisition photons of up to 20,000 or more. SymphoTime 64 software (PicoQuant, Germany) was used to calculate the decay curves per pixel and fitted with a decay model. Double-exponential was selected for the test combination with donor YFP-PdbUBC34s and receptor PdbMYB221-RFP, and the mono-exponential model was applied for only donor YFP-PdbUBC34s as a control.

### Transaction assays

The vectors CaMV *35S*-*GAL4DB*-*SUPRD* and CaMV *35S*-*GAL4*-*TATA*-*Ω*-*LUC*-*Nos* were gifts from Prof. Masaru Ohme-Takagi (National Institute of Advanced Industrial Technology and Science, Tokyo, Japan) [47], from which the regions of *GAL4DB*-*SUPRD* and *GAL4*-*TATA*-*Ω* were amplified with the primers (Table S1) and cloned into the binary vectors of pGreenII 62-SK and pGreenII 0800-LUC (Fig. S6i), respectively, producing the effector vector and the reporter vector. The expression cassette of *Renilla* luciferase (RLuc) was also included in the pGreenII 0800-LUC vector, serving as an internal control. The transaction assays were performed with the Dual-Luciferase Reporter Assay System (#E1980, Promega) according to the manufacturer’s protocol. The LUC expression values of each transformation were normalized to the RLuc values. Each data point represents at least eight replications. Three independent experiments were performed.

### Induction of secondary walls

The coding regions of three key activators of SCW biosynthesis, namely *VNS07/WND6A*, *VNS09/WND2A*, and *MYB020*, were amplified from *P. davidiana* × *bolleana* cDNA with the primers (Table S1) and cloned into the binary vector pGreenII 62-SK (Fig. S6i) for transient overexpression in poplar leaves. The vector pGreenII 62-SK was used as a negative control. The transiently transformed leaves were stained with basic fuchsin at 10 dpi and observed for secondary wall deposition with a confocal microscope as described previously [75].

### Generation of the stably transformed poplar

The infiltrated leaves with *Agrobacterium* GV3101 harboring the binary plasmid pSuper1300-*Super*:*GFP* (Fig. S6c) with hygromycin resistance or CaMV*35S*:*GUS*-intro (Fig. S6b) with kanamycin resistance were harvested from plants at 9 dpi. These leaves were washed thoroughly under running tap water, sterilized for 10 min in 2% sodium hypochlorite solution supplemented with 0.01% Tween 20, and rinsed three times with sterile water. The infiltrated part of the leaves, marked at the time of infiltration, was cut into pieces with caution to avoid the midrib. The transformed plants were regenerated via callus-induced indirect organogenesis. First, the callus induction was performed on MS medium supplemented with 1 mg/L 2,4-D, 0.1 mg/L NAA, 0.2 mg/ L 6-BA, 0.01 mg/L TDZ, 0.1 g/L Tim, 0.1 g/L Cefo, and 4.5 mg/L hygromycin (for GFP transformants) or 50 mg/L kanamycin (for GUS transformants) at 25 °C in the dark for 3–4 weeks. Then, the calli were transferred to shooting medium [MS medium supplemented with 0.1 mg/L NAA, 0.2 mg/ L 6-BA, 0.01 mg/L TDZ, 0.1 g/L Tim, 0.1 g/L Cefo, and 4.5 mg/L hygromycin (for GFP transformants) or 50 mg/L kanamycin (for GUS transformants)] and cultured at 25 °C with a 16 h light/8 h light/dark cycle. These calli were sub-cultured onto fresh shooting medium every 2 weeks until shoots formed. Shoots were excised at about 1.0 cm down from the apical tip and cultured on rooting medium (MS medium supplemented with 0.1 mg/L NAA, 0.1 g/L Tim, 0.1 g/L Cefo, and 4.5 mg/L hygromycin (for GFP transformants) or 50 mg/L kanamycin (for GUS transformants)) at 25 °C and 16 h light/8 h light/dark cycles. GFP positive calli were verified under a DFP-1 Dual Fluorescent Protein Flashlight (Night Sea, USA) before they were transferred for shoot regeneration. The calli introduced with *GUS* were stained for GUS after the regenerated shoots were excised. The rooting regenerants in which the roots appeared within 5–10 days after being transferred into rooting medium were checked for reporters GFP or GUS expression. The transformation via direct organogenesis was conducted as described previously [63].

GFP fluorescent signals in the transformed callus and intact plants expressing the GFP reporter were observed using a DFP-1 Dual Fluorescent Protein Flashlight (Night Sea, USA). The plant materials were illuminated by RB-Royal Blue (400–460 nm) and observed and photographed through a yellow filter.

### Microscopy

Poplar leaves LPI 4, with an exception of leaves LPI 3 of *P. trichocarpa*, were used for investigating the inner structure of the leaves. The transverse sections of the leaves were prepared as described earlier [76]. Five-micrometer-thick sections were cut with a microtome (Leica RM2265, Germany), stained with toluidine blue O (TBO) as described previously [68], and observed using a Leica DM 5500 B light microscope (Leica, Germany).

## Supplementary Information

**Additional file 1 Fig. S1** The transient expression efficiency of the LUC reporter in clones *P. davidiana* × *bolleana*, *P. alba var. pyramidalis*, and *P. trichocarpa*. **Fig. S2** The effect of *Agrobacterium* strains and Acetosyringone (AS) concentration in the infiltrated medium on transient expression from the initial experiments. **Fig. S3** The effect of the bacterial growth stage and density, infiltration medium on the transient expression efficiency in poplar leaves. **Fig. S4** Generation of stably transformed plants from transient transformed leaves. **Fig. S5** Callus induction in leaf explants during direct organogenesis and the callus-induced indirect organogenesis process. **Fig. S6** Schematic representation of T-DNA regions of the constructs used in this study. **Fig. S7** Uncropped images of co-immunoprecipitation assay shown in Fig. 5c. **Fig. S8** Uncropped images of the immunoblot assay shown in Fig. S2.

**Additional file 2 Table S1** Primer list for vector construct.

## Data Availability

All data generated or analyzed during this study are included in this published article and its supplementary information files. All materials used in this study are available from the corresponding author. The accession numbers of genes used in this study are as follows: PdbCBL1 (MN400431), PdbMTP1 (MN400432), PdbC4H (MN400430), PdbGT47C (MN400434), PdbPrxQ (MN400433), PdbVNS07 (MN887349), PdbVNS09 (MN887350), PdbMYB020 (MN887351), AtWRKY40 (AT1G80840), AtNRT2.1 (AT1G08090), AtNRT3.1 (AT5G50200), and PtoUBC34 (MH708242).

## Abbreviations

2,4-D: 2,4-Dichlorophenoxyacetic acid
6-BA: 6-Benzylaminopurine
BiFC: Bimolecular fluorescence complementation
CaMV 35S promoter: Cauliflower mosaic virus 35S promoter
Cefo: Cefotaxime
CoIP: Coinmunoprecipitation
ER: Endoplasmic reticulum
FRET-FLIM: Förster resonance energy transfer measured by fluorescence lifetime microscopy
LPI: Leaf plastochron index
LUC: Luciferase
NAA: Naphthalene-acetic acid
OD: Optical density
PCR: Polymerase chain reaction
PM: Plasma membrane
PI: Plastochron index
RFP: Red fluorescent protein
RLuc: Renilla luciferase
SCW: Secondary cell wall
SUPRD: The EAR-like motif repression domain of SUPERMAN
Tim: Timentin
TDZ: Thidiazuron
