# Supplementary material for: An improved and efficient method of Agrobacterium syringe infiltration for transient transformation and its application in the elucidation of gene function in poplar
Source: BMC Plant Biol. 2021 Jan 21;21:54. doi: 10.1186/s12870-021-02833-w (PMC7818742; doi:10.1186/s12870-021-02833-w)
Supplement: Supplementary file 1 — Additional file 1 Fig. S1 The transient expression efficiency of the LUC reporter in clones P. davidiana × bolleana, P. alba var. pyramidalis, and P. trichocarpa. Fig. S2 The effect of Agrobacterium strains and Acetosyringone (AS) concentration in the infiltrated medium on transient expression from the initial experiments. Fig. S3 The effect of the bacterial growth stage and density, infiltration medium on the transient expression efficiency in poplar leaves. Fig. S4 Generation of stably transformed plants from transient transformed leaves. Fig. S5 Callus induction in leaf explants during direct organogenesis and the callus-induced indirect organogenesis process. Fig. S6 Schematic representation of T-DNA regions of the constructs used in this study. Fig. S7 Uncropped images of co-immunoprecipitation assay shown in Fig. 5c. Fig. S8 Uncropped images of the immunoblot assay shown in Fig. S2. [file 12870_2021_2833_MOESM1_ESM.pptx]

## Slide 1
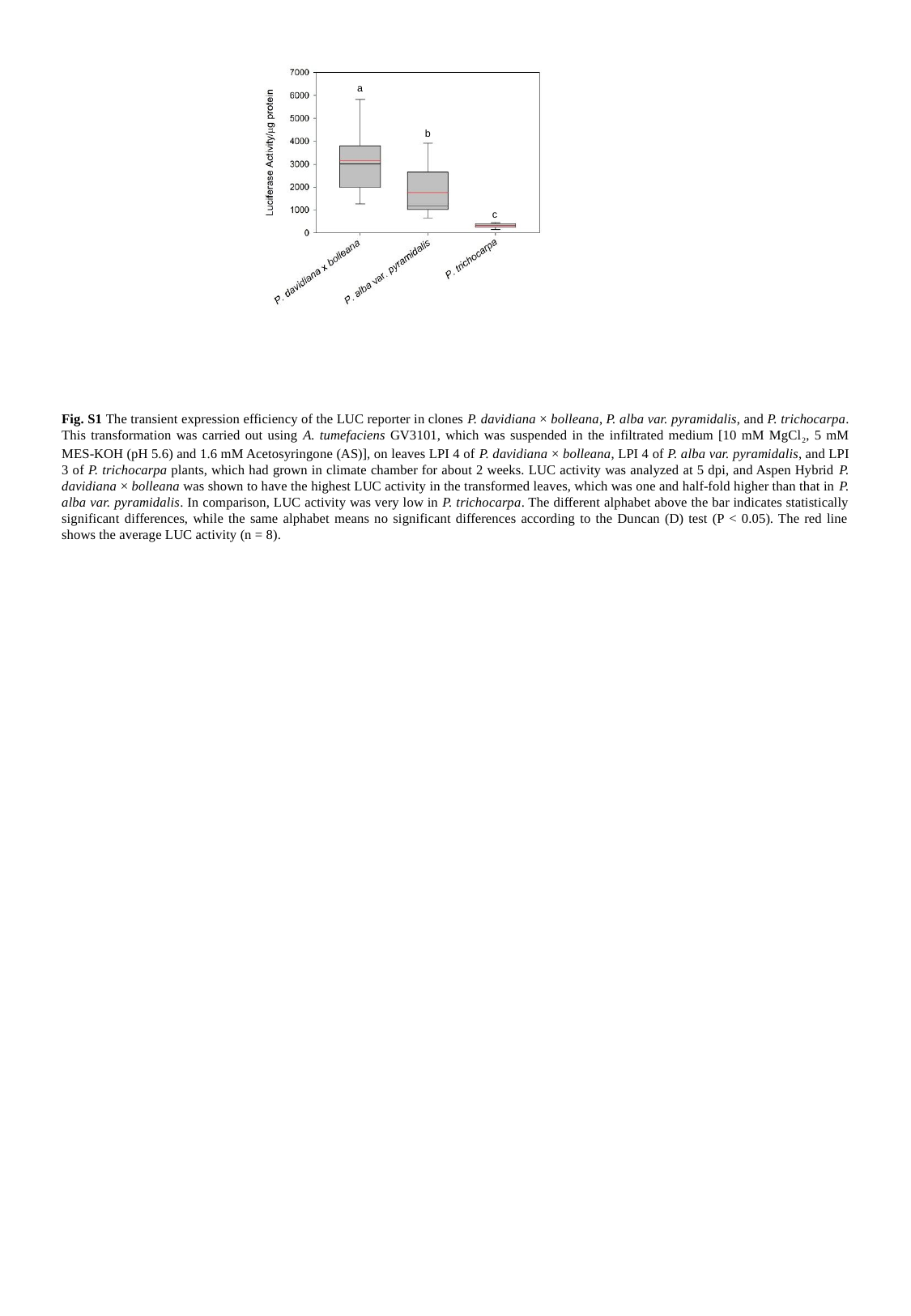

a
b
c
Fig. S1 The transient expression efficiency of the LUC reporter in clones P. davidiana × bolleana, P. alba var. pyramidalis, and P. trichocarpa. This transformation was carried out using A. tumefaciens GV3101, which was suspended in the infiltrated medium [10 mM MgCl2, 5 mM MES-KOH (pH 5.6) and 1.6 mM Acetosyringone (AS)], on leaves LPI 4 of P. davidiana × bolleana, LPI 4 of P. alba var. pyramidalis, and LPI 3 of P. trichocarpa plants, which had grown in climate chamber for about 2 weeks. LUC activity was analyzed at 5 dpi, and Aspen Hybrid P. davidiana × bolleana was shown to have the highest LUC activity in the transformed leaves, which was one and half-fold higher than that in P. alba var. pyramidalis. In comparison, LUC activity was very low in P. trichocarpa. The different alphabet above the bar indicates statistically significant differences, while the same alphabet means no significant differences according to the Duncan (D) test (P < 0.05). The red line shows the average LUC activity (n = 8).

## Slide 2
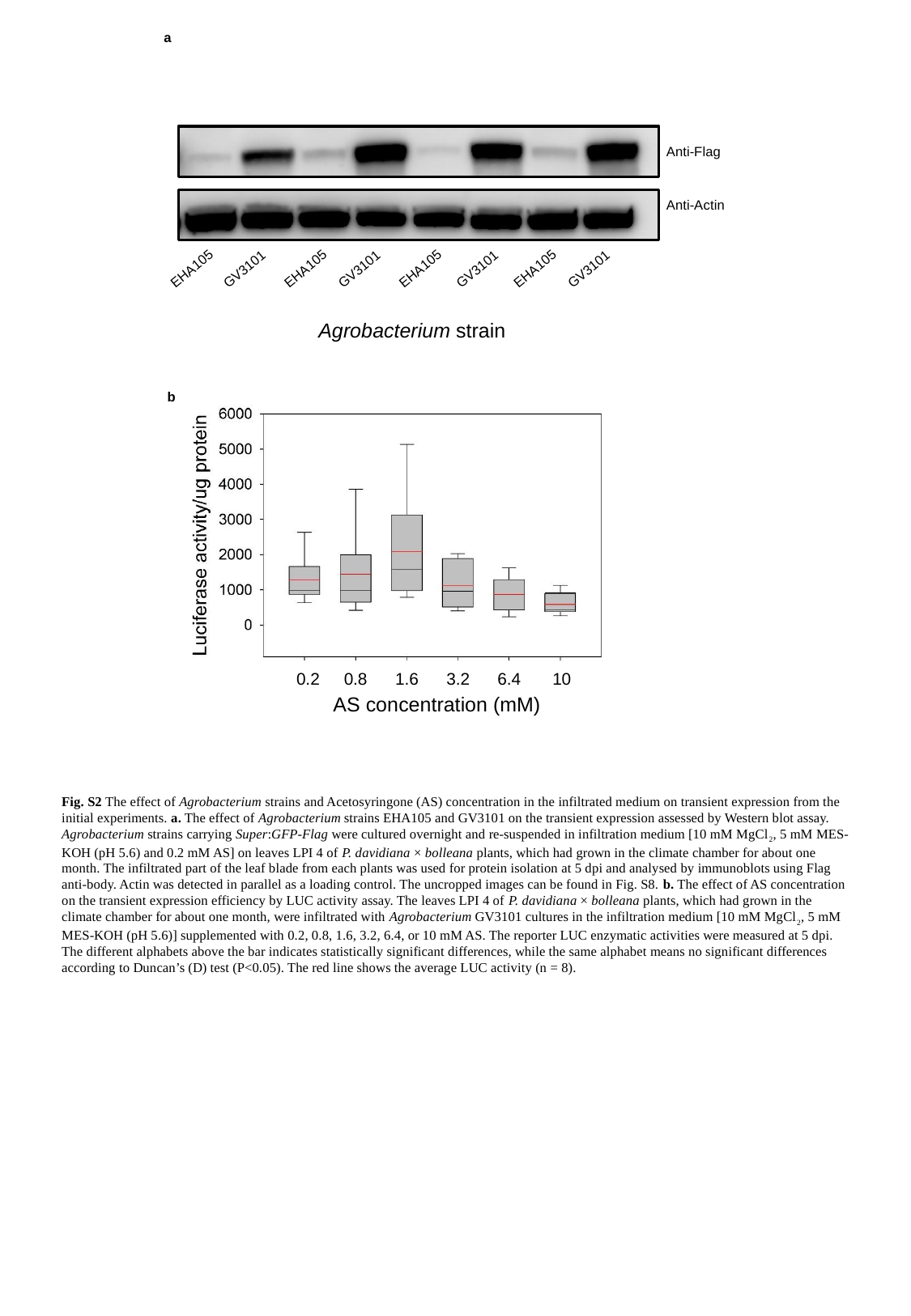

a
Anti-Flag
Anti-Actin
GV3101
GV3101
EHA105
GV3101
GV3101
EHA105
EHA105
EHA105
Agrobacterium strain
b
0.2
0.8
1.6
3.2
6.4
10
AS concentration (mM)
Fig. S2 The effect of Agrobacterium strains and Acetosyringone (AS) concentration in the infiltrated medium on transient expression from the initial experiments. a. The effect of Agrobacterium strains EHA105 and GV3101 on the transient expression assessed by Western blot assay. Agrobacterium strains carrying Super:GFP-Flag were cultured overnight and re-suspended in infiltration medium [10 mM MgCl2, 5 mM MES-KOH (pH 5.6) and 0.2 mM AS] on leaves LPI 4 of P. davidiana × bolleana plants, which had grown in the climate chamber for about one month. The infiltrated part of the leaf blade from each plants was used for protein isolation at 5 dpi and analysed by immunoblots using Flag anti-body. Actin was detected in parallel as a loading control. The uncropped images can be found in Fig. S8. b. The effect of AS concentration on the transient expression efficiency by LUC activity assay. The leaves LPI 4 of P. davidiana × bolleana plants, which had grown in the climate chamber for about one month, were infiltrated with Agrobacterium GV3101 cultures in the infiltration medium [10 mM MgCl2, 5 mM MES-KOH (pH 5.6)] supplemented with 0.2, 0.8, 1.6, 3.2, 6.4, or 10 mM AS. The reporter LUC enzymatic activities were measured at 5 dpi. The different alphabets above the bar indicates statistically significant differences, while the same alphabet means no significant differences according to Duncan’s (D) test (P<0.05). The red line shows the average LUC activity (n = 8).

## Slide 3
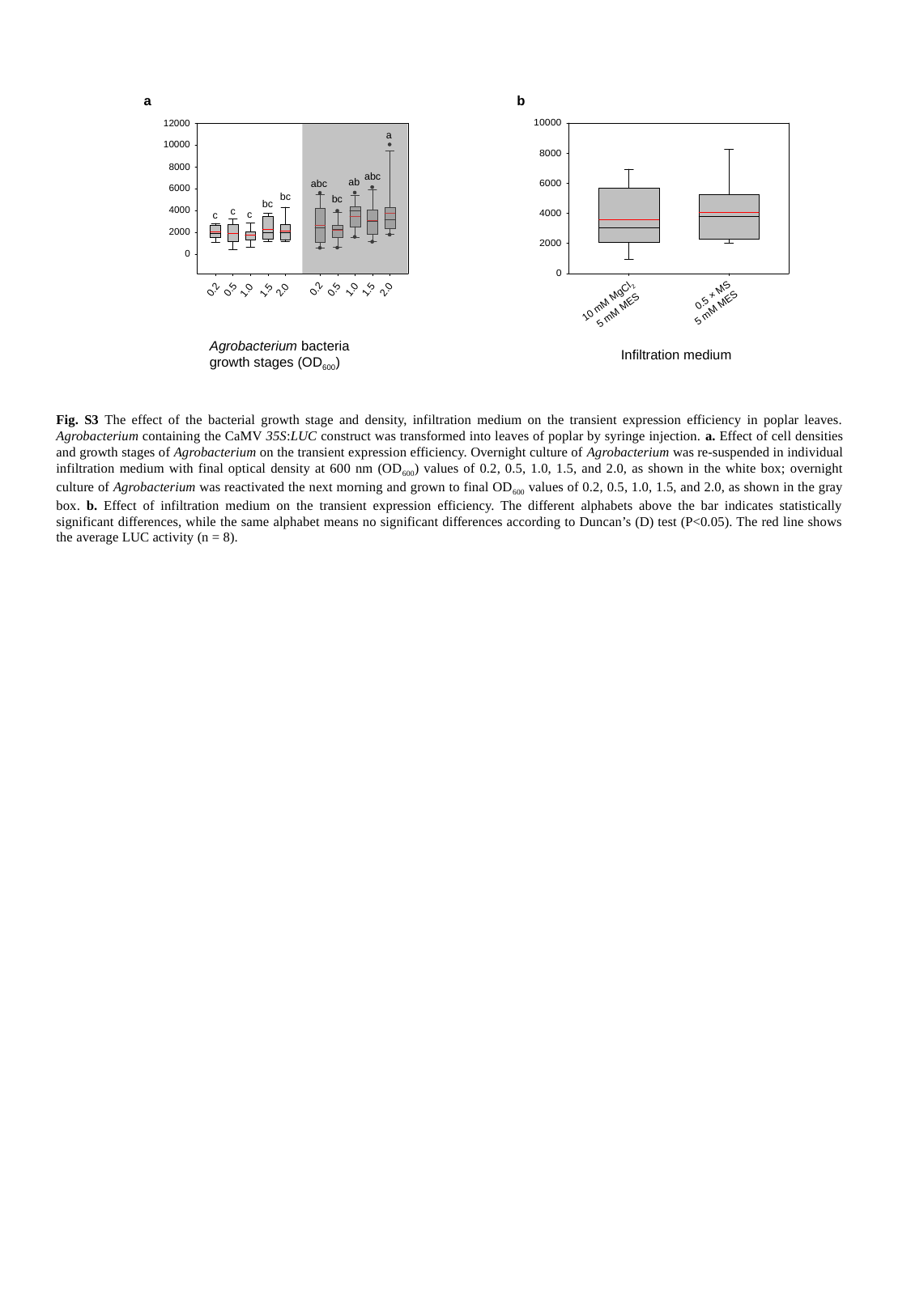

a
b
B
a
abc
ab
abc
bc
bc
bc
c
c
c
0.2
2.0
0.2
0.5
1.0
0.5
1.5
2.0
1.0
1.5
0.5 × MS
5 mM MES
10 mM MgCl2
5 mM MES
Agrobacterium bacteria
growth stages (OD600)
 Infiltration medium
Fig. S3 The effect of the bacterial growth stage and density, infiltration medium on the transient expression efficiency in poplar leaves. Agrobacterium containing the CaMV 35S:LUC construct was transformed into leaves of poplar by syringe injection. a. Effect of cell densities and growth stages of Agrobacterium on the transient expression efficiency. Overnight culture of Agrobacterium was re-suspended in individual infiltration medium with final optical density at 600 nm (OD600) values of 0.2, 0.5, 1.0, 1.5, and 2.0, as shown in the white box; overnight culture of Agrobacterium was reactivated the next morning and grown to final OD600 values of 0.2, 0.5, 1.0, 1.5, and 2.0, as shown in the gray box. b. Effect of infiltration medium on the transient expression efficiency. The different alphabets above the bar indicates statistically significant differences, while the same alphabet means no significant differences according to Duncan’s (D) test (P<0.05). The red line shows the average LUC activity (n = 8).

## Slide 4
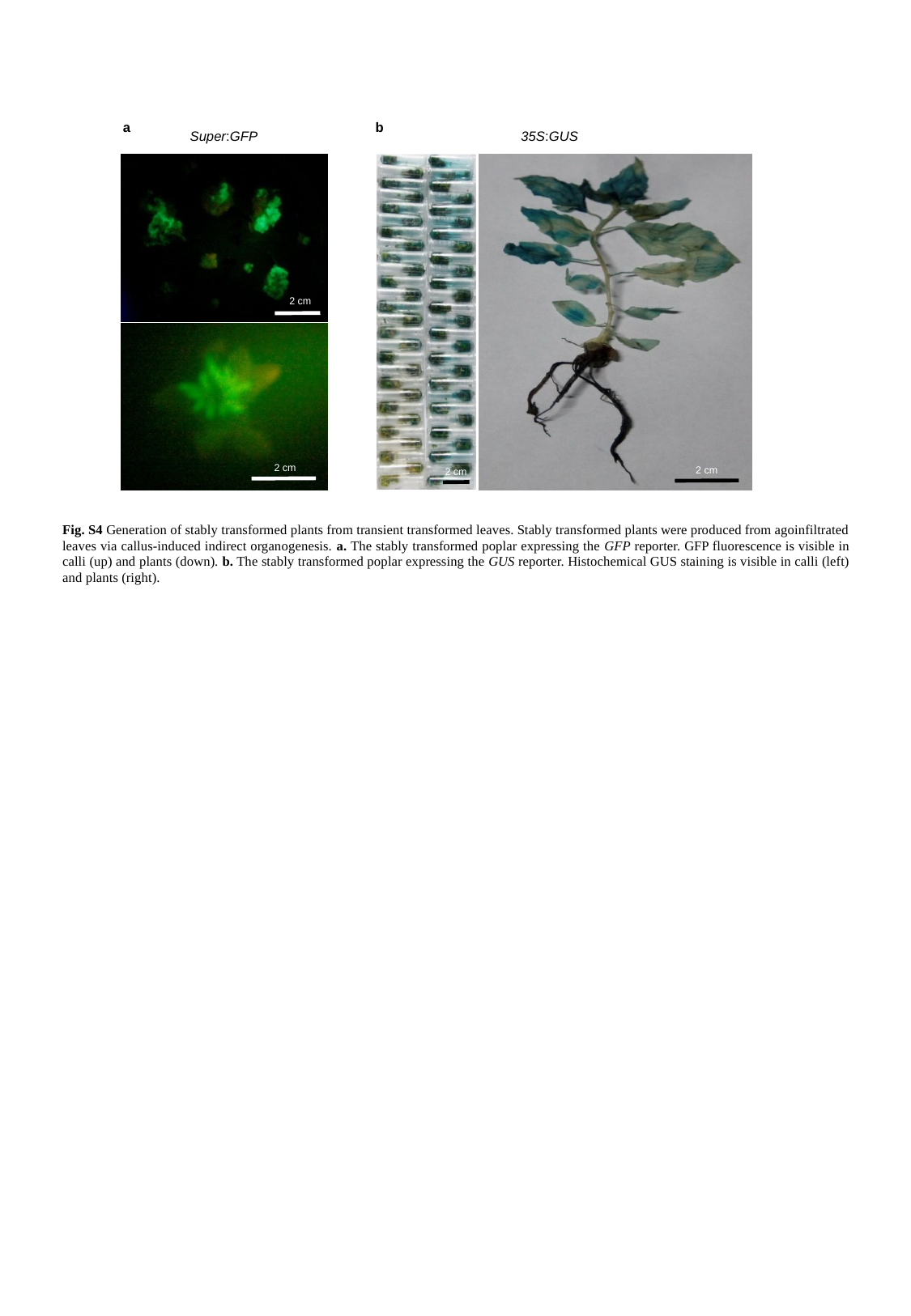

a
b
Super:GFP
35S:GUS
2 cm
2 cm
2 cm
2 cm
Fig. S4 Generation of stably transformed plants from transient transformed leaves. Stably transformed plants were produced from agoinfiltrated leaves via callus-induced indirect organogenesis. a. The stably transformed poplar expressing the GFP reporter. GFP fluorescence is visible in calli (up) and plants (down). b. The stably transformed poplar expressing the GUS reporter. Histochemical GUS staining is visible in calli (left) and plants (right).

## Slide 5
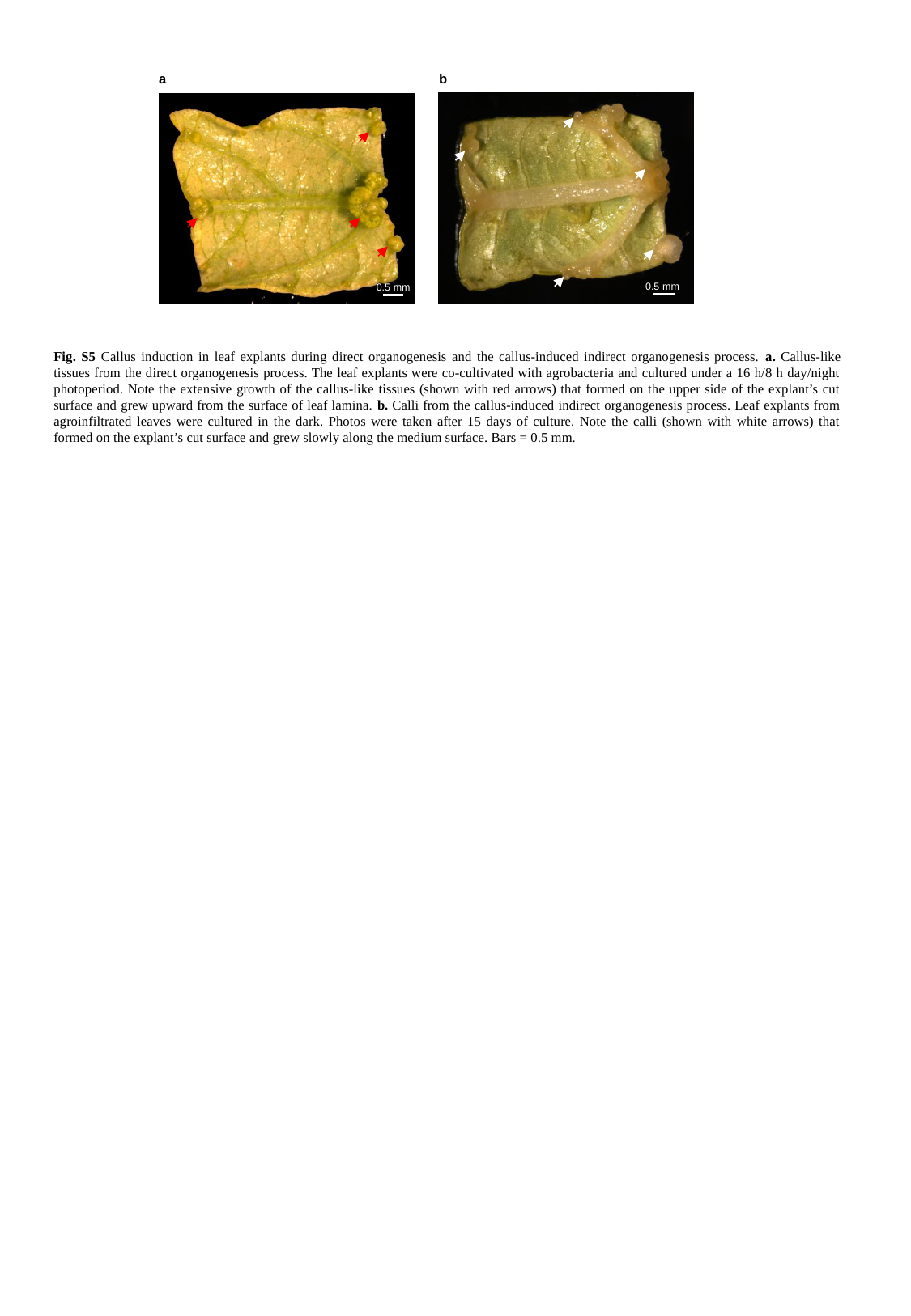

a
b
0.5 mm
0.5 mm
Fig. S5 Callus induction in leaf explants during direct organogenesis and the callus-induced indirect organogenesis process. a. Callus-like tissues from the direct organogenesis process. The leaf explants were co-cultivated with agrobacteria and cultured under a 16 h/8 h day/night photoperiod. Note the extensive growth of the callus-like tissues (shown with red arrows) that formed on the upper side of the explant’s cut surface and grew upward from the surface of leaf lamina. b. Calli from the callus-induced indirect organogenesis process. Leaf explants from agroinfiltrated leaves were cultured in the dark. Photos were taken after 15 days of culture. Note the calli (shown with white arrows) that formed on the explant’s cut surface and grew slowly along the medium surface. Bars = 0.5 mm.

## Slide 6
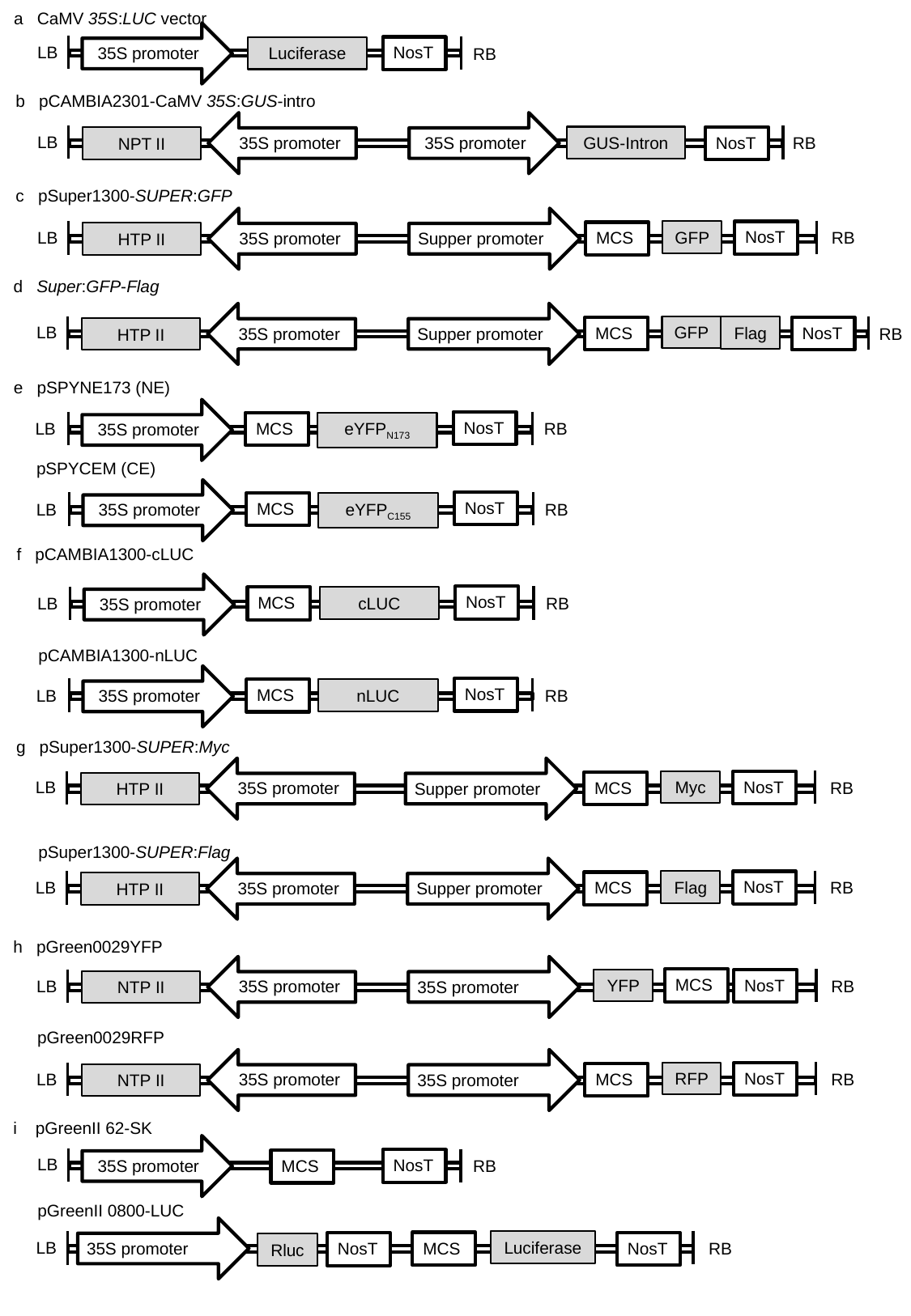

a CaMV 35S:LUC vector
35S promoter
LB
NosT
Luciferase
RB
b pCAMBIA2301-CaMV 35S:GUS-intro
35S promoter
35S promoter
LB
RB
NosT
GUS-Intron
NPT II
c pSuper1300-SUPER:GFP
35S promoter
Supper promoter
NosT
GFP
LB
MCS
RB
HTP II
d Super:GFP-Flag
35S promoter
Supper promoter
GFP
LB
NosT
Flag
MCS
RB
HTP II
e pSPYNE173 (NE)
35S promoter
NosT
MCS
LB
eYFPN173
RB
pSPYCEM (CE)
35S promoter
NosT
MCS
eYFPC155
RB
LB
f pCAMBIA1300-cLUC
35S promoter
NosT
MCS
LB
cLUC
RB
35S promoter
NosT
MCS
LB
nLUC
RB
pCAMBIA1300-nLUC
g pSuper1300-SUPER:Myc
35S promoter
Supper promoter
NosT
Myc
LB
MCS
RB
HTP II
pSuper1300-SUPER:Flag
35S promoter
Supper promoter
NosT
Flag
LB
MCS
RB
HTP II
h pGreen0029YFP
35S promoter
35S promoter
MCS
NosT
YFP
LB
RB
NTP II
pGreen0029RFP
35S promoter
35S promoter
NosT
RFP
LB
MCS
RB
NTP II
i pGreenII 62-SK
35S promoter
LB
NosT
MCS
RB
pGreenII 0800-LUC
35S promoter
Luciferase
LB
MCS
RB
NosT
NosT
Rluc

## Slide 7
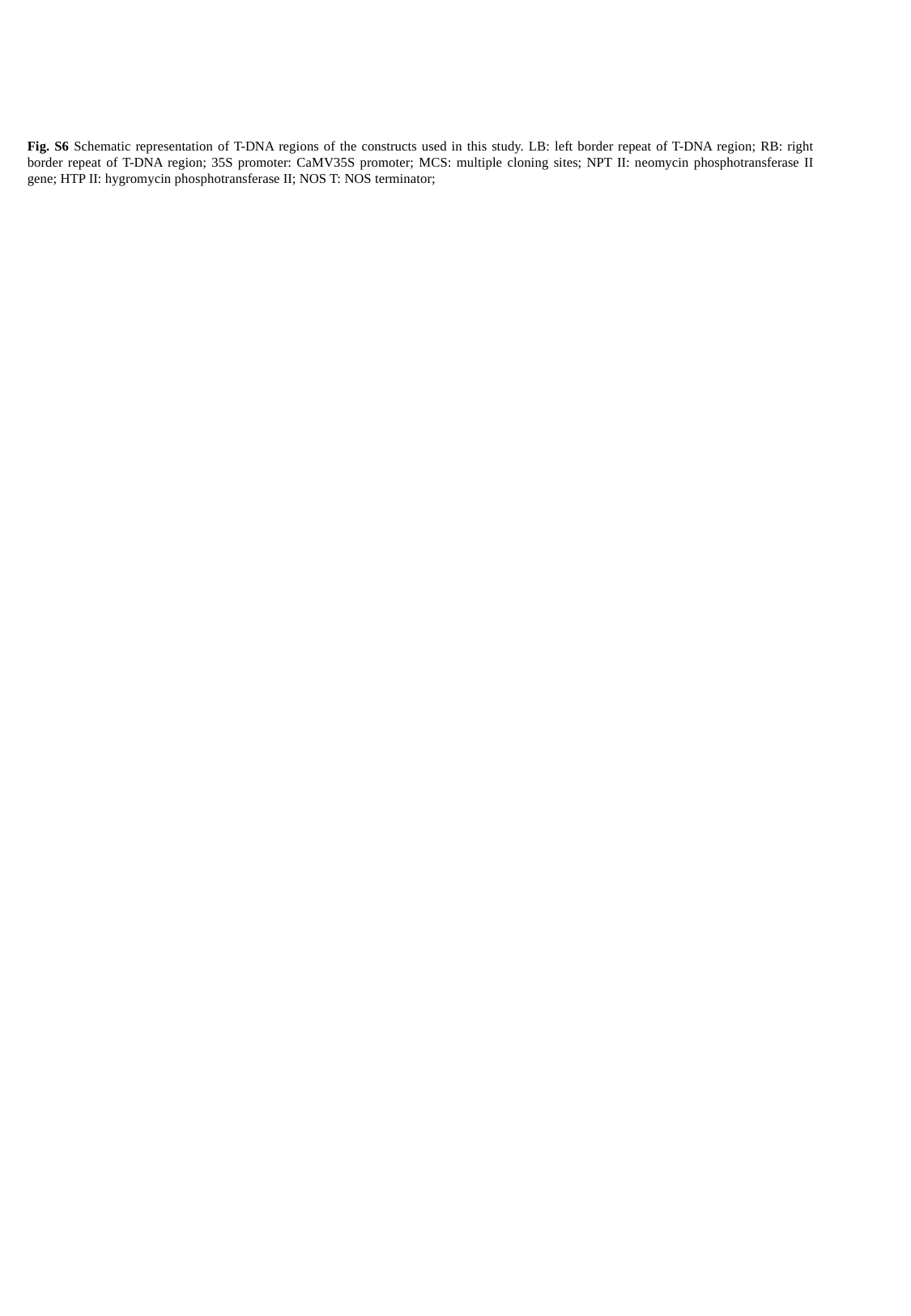

Fig. S6 Schematic representation of T-DNA regions of the constructs used in this study. LB: left border repeat of T-DNA region; RB: right border repeat of T-DNA region; 35S promoter: CaMV35S promoter; MCS: multiple cloning sites; NPT II: neomycin phosphotransferase II gene; HTP II: hygromycin phosphotransferase II; NOS T: NOS terminator;

## Slide 8
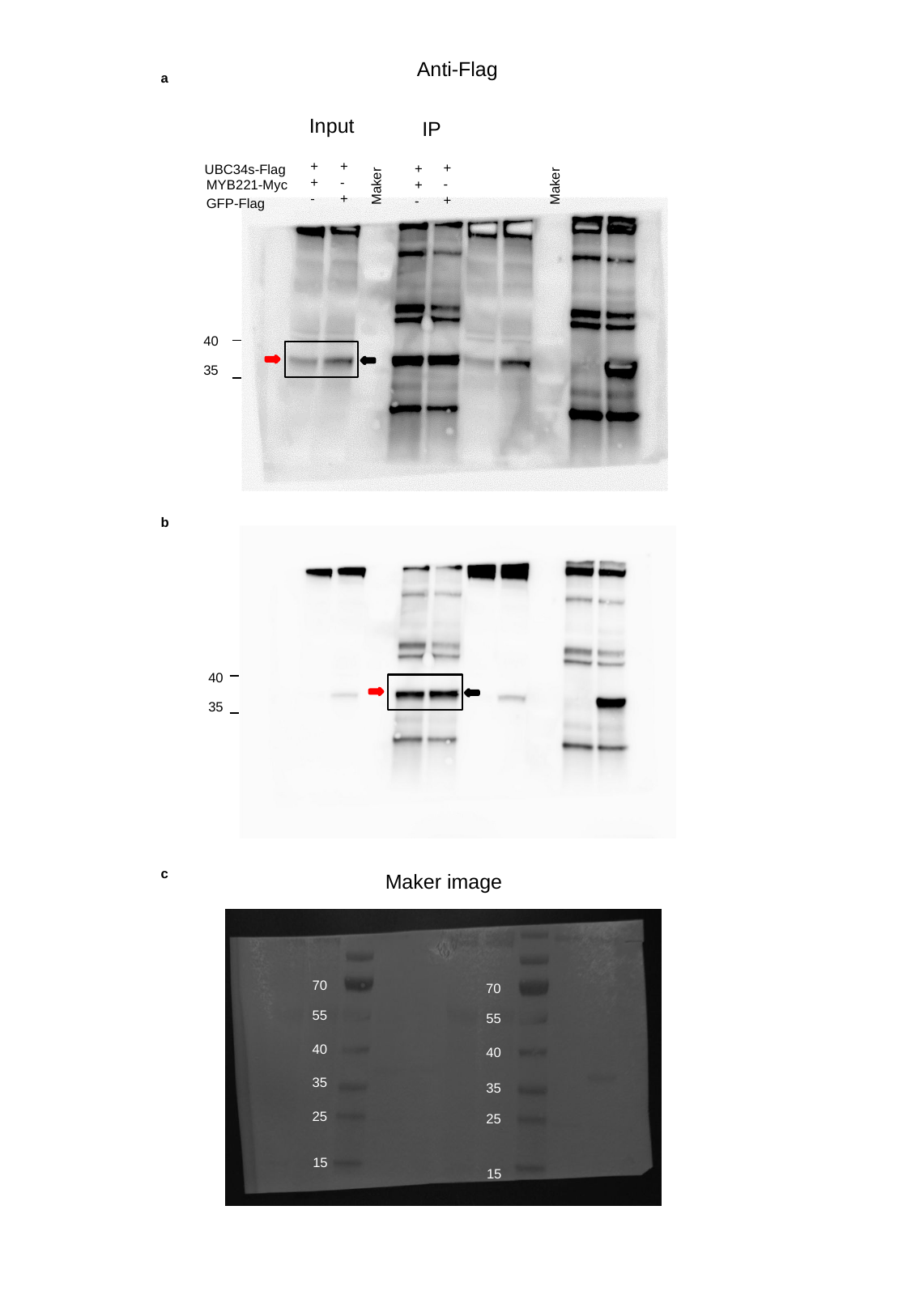

Anti-Flag
Input
IP
Maker
+
-
+
+
+
-
+
-
+
+
+
-
UBC34s-Flag
Maker
MYB221-Myc
GFP-Flag
40
35
40
35
Maker image
70
70
55
55
40
40
35
35
25
25
15
15
a
b
c

## Slide 9
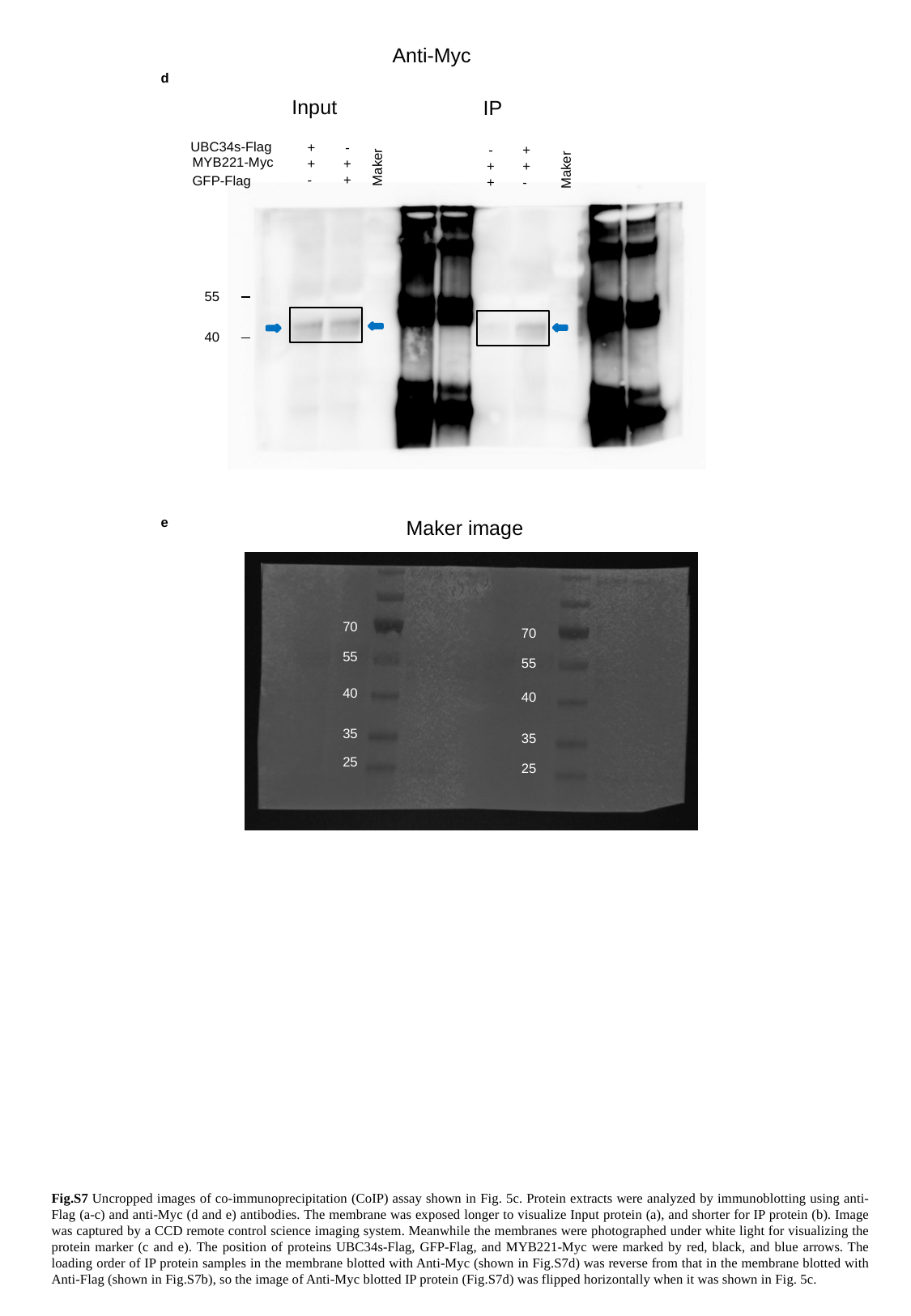

Anti-Myc
Input
IP
-
+
+
Maker
-
+
+
Maker
UBC34s-Flag
+
+
-
+
+
-
MYB221-Myc
GFP-Flag
55
40
d
e
Maker image
70
70
55
55
40
40
35
35
25
25
Fig.S7 Uncropped images of co-immunoprecipitation (CoIP) assay shown in Fig. 5c. Protein extracts were analyzed by immunoblotting using anti-Flag (a-c) and anti-Myc (d and e) antibodies. The membrane was exposed longer to visualize Input protein (a), and shorter for IP protein (b). Image was captured by a CCD remote control science imaging system. Meanwhile the membranes were photographed under white light for visualizing the protein marker (c and e). The position of proteins UBC34s-Flag, GFP-Flag, and MYB221-Myc were marked by red, black, and blue arrows. The loading order of IP protein samples in the membrane blotted with Anti-Myc (shown in Fig.S7d) was reverse from that in the membrane blotted with Anti-Flag (shown in Fig.S7b), so the image of Anti-Myc blotted IP protein (Fig.S7d) was flipped horizontally when it was shown in Fig. 5c.

## Slide 10
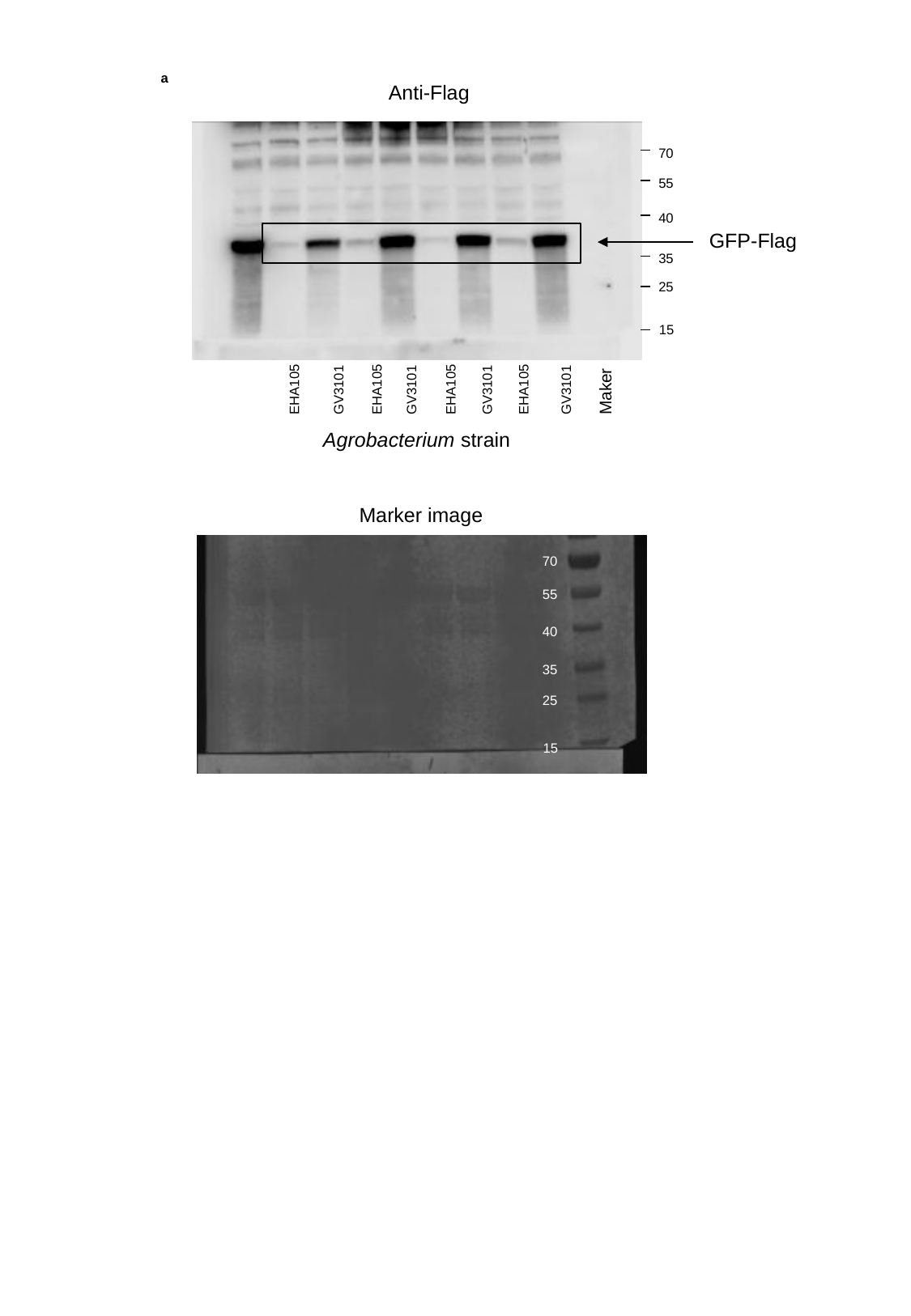

a
Anti-Flag
EHA105
GV3101
EHA105
GV3101
EHA105
GV3101
EHA105
GV3101
Maker
Agrobacterium strain
70
55
40
GFP-Flag
35
25
15
Marker image
70
55
40
35
25
15

## Slide 11
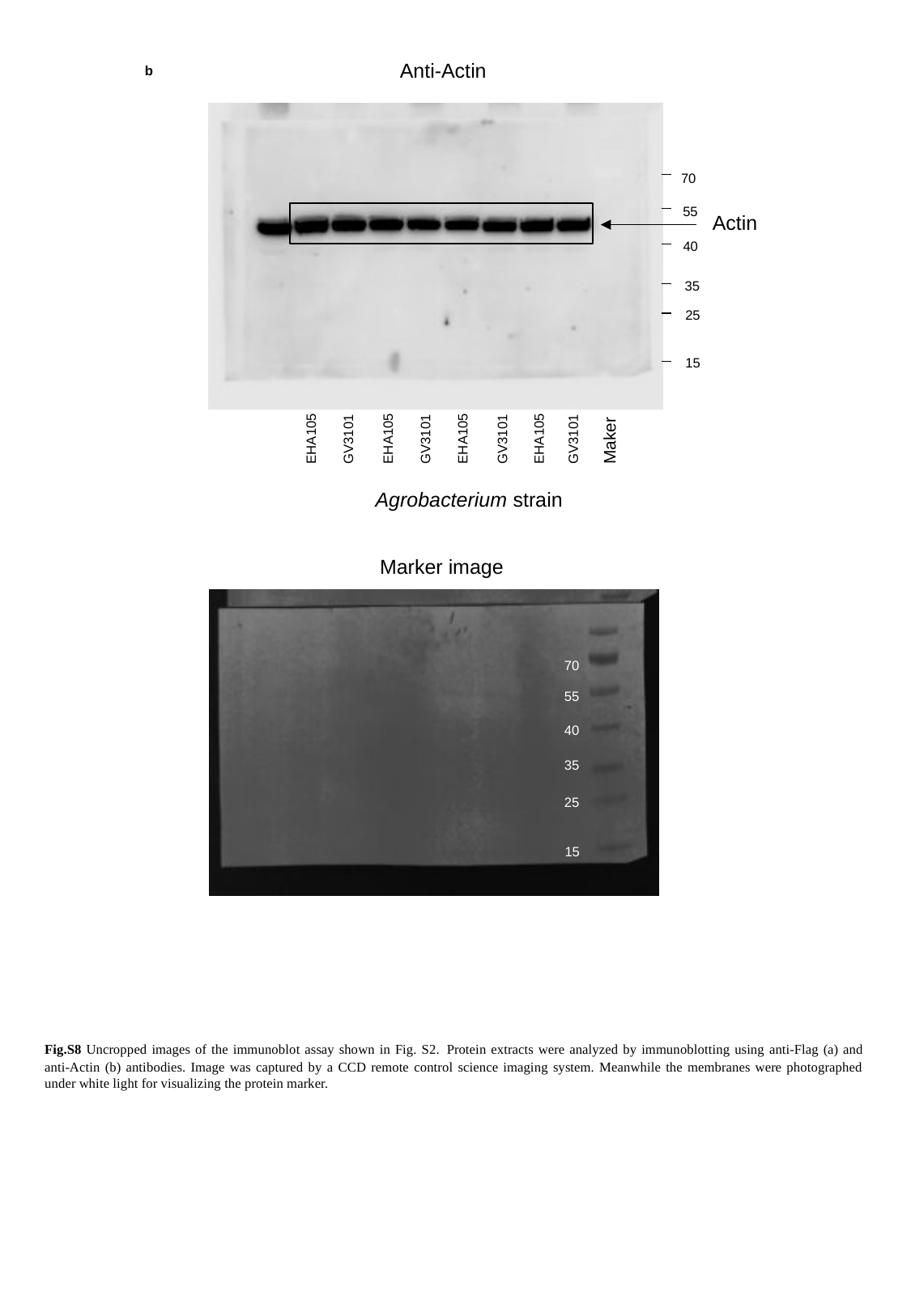

Anti-Actin
70
55
Actin
40
35
25
15
EHA105
GV3101
EHA105
GV3101
EHA105
GV3101
EHA105
GV3101
Maker
Agrobacterium strain
b
Marker image
70
55
40
35
25
15
Fig.S8 Uncropped images of the immunoblot assay shown in Fig. S2. Protein extracts were analyzed by immunoblotting using anti-Flag (a) and anti-Actin (b) antibodies. Image was captured by a CCD remote control science imaging system. Meanwhile the membranes were photographed under white light for visualizing the protein marker.
