# Supplementary material for: An improved and efficient method of Agrobacterium syringe infiltration for transient transformation and its application in the elucidation of gene function in poplar
Source: BMC Plant Biol. 2021 Jan 21;21:54. doi: 10.1186/s12870-021-02833-w (PMC7818742; doi:10.1186/s12870-021-02833-w)
Supplement: Supplementary file 2 — Additional file 2 Table S1 Primer list for vector construct. [file 12870_2021_2833_MOESM2_ESM.docx]

**Table S1 Primer list for vector construct**

| **Name** | **Sequence** | | | **Notes** |
| --- | --- | --- | --- | --- |
| **Subcellular localization**  PdbPrxQ-F  PdbPrxQ-R  PdbMPT1-F  PdbMPT1-R  PdbGT47C-F  PdbGT47C-R  PdbCBL1-F  PdbCBL1-R  PdbC4H- F  PdbC4H- R  PdbMYB221-F  PdbMYB221-R  **BiFC assay**  AtWRKY40-F  AtWRKY40-R  **Transactivation assays**  GAL4DB-SUPRD-F  GAL4DB-SUPRD-R  GAL4-F  GAL4-R  **Induction of SCW**  PdbVNS7-F  PdbVNS7-R  PdbVNS9-F  PdbVNS9-R  PdbMYB20-F  PdbMYB20-R  VP16-F  VP16-R | | CTGCAGATGGCTTCCATTTCTCTCC  GGTACCAAGGCTTTGAAGTAGTTTAAGA  CTGCAGATGGAAGCACAAAATCCTC  GGTACCACGCTCTATCTGGATGGTTAC  CTGCAGATGGTACTGGAGGTTAAAAGAC  GGTACCGTGGTTTGAAAGCCTCACG  CTGCAGATGGGCTGTTTTAGTTCCA  GGTACCTGAAGCAATCTCATCCACC  CTGCAGATGGATCTCCTCCTCCTG  ACTAGTAAAGGACCTTGGCTTTG  GTCGACATGGGAAGGTCTCCTTGCT  GGTACCTTTCATCTCCAAACCTCTA  ACTAGTATGGATCAGTACTCATCCTCTT  CTCGAGTTTCTCGGTATGATTCTGTTG  ACTAGTATGAAGCTACTGTCTTCTATCG  GAATTCTTAAGCGAAACCCAAAC  CCCGGGAAGCTTGCATGCCTGC  ACTAGTGGTCGACTGTAATTGTAAAAG  GGATCCATGGAATCCTGTGTCCCACC  GTCGACTATGTCAGGAAAGCAGTCAAGG  GGATCCATGACAGAAAACATGAGTATATCTG  GTCGACTGCACCCGTGTCTGACAA  GGATCCATGAGGAAGCCGGATCTAGT  GTCGACTTCTACGTGGAAATCATGTAAAG  GTCGACGCCCCCCCGACCGATGTCAGCC  GGTACCCTACCCACCGTACTCGTCAAT | **Restriction enzyme used**  Pst I  Kpn I  Pst I  Kpn I  Pst I  Kpn I  Pst I  Kpn I  Pst I  Spe I  Sal I  Kpn I  Spe I  Xho I  Spe I  EcoR I  Sma I  Spe I  BamH I  Sal I  BamH I  Sal I  BamH I  Sal I  Sal I  Kpn I | |

Underlined letters represent restriction enzyme recognition sites.
